# Compression force sensing regulates integrin α_IIb_β_3_ adhesive function on diabetic platelets

**DOI:** 10.1038/s41467-018-03430-6

**Published:** 2018-03-14

**Authors:** Lining Ju, James D. McFadyen, Saheb Al-Daher, Imala Alwis, Yunfeng Chen, Lotte L. Tønnesen, Sophie Maiocchi, Brianna Coulter, Anna C. Calkin, Eric I. Felner, Neale Cohen, Yuping Yuan, Simone M. Schoenwaelder, Mark E. Cooper, Cheng Zhu, Shaun P. Jackson

**Affiliations:** 1 Heart Research Institute, Thrombosis Group, Newtown, New South Wales, 2042 Australia; 2 Charles Perkins Centre, Level 3E Cardiovascular Division, The University of Sydney, New South Wales, 2006 Australia; 3 Australian Centre for Blood Diseases, Central Clinical School, Monash University, Melbourne, Victoria 3004 Australia; 4 Coulter Department of Biomedical Engineering; and Woodruff School of Mechanical Engineering, Georgia Institute of Technology, Atlanta, GA 30332 USA; 5 Parker H. Petit Institute for Bioengineering and Bioscience, Georgia Institute of Technology, Atlanta, GA 30332 USA; 6 Department of Molecular and Experimental Medicine, The Scripps Research Institute, La Jolla, 92037 CA USA; 7 Lipid Metabolism and Cardiometabolic Disease Laboratory, Baker Heart and Diabetes Institute, Melbourne, Victoria, 3004 Australia; 8 Division of Pediatric Endocrinology, Emory University School of Medicine, Atlanta, GA 30322 USA; 9 Clinical Diabetes, Baker Heart and Diabetes Institute, Melbourne, Victoria 3004 Australia; 10 Department of Diabetes, Central Clinical School, Monash University, Melbourne, 3004 Victoria Australia

## Abstract

Diabetes is associated with an exaggerated platelet thrombotic response at sites of vascular injury. Biomechanical forces regulate platelet activation, although the impact of diabetes on this process remains ill-defined. Using a biomembrane force probe (BFP), we demonstrate that compressive force activates integrin α_IIb_β_3_ on discoid diabetic platelets, increasing its association rate with immobilized fibrinogen. This compressive force-induced integrin activation is calcium and PI 3-kinase dependent, resulting in enhanced integrin affinity maturation and exaggerated shear-dependent platelet adhesion. Analysis of discoid platelet aggregation in the mesenteric circulation of mice confirmed that diabetes leads to a marked enhancement in the formation and stability of discoid platelet aggregates, via a mechanism that is not inhibited by therapeutic doses of aspirin and clopidogrel, but is eliminated by PI 3-kinase inhibition. These studies demonstrate the existence of a compression force sensing mechanism linked to α_IIb_β_3_ adhesive function that leads to a distinct prothrombotic phenotype in diabetes.

## Abstract (web summary)

Diabetes is associated with an increased thrombotic response, but the mechanism is unknown. Here the authors demonstrate that compressive force activates integrin α_IIb_β_3_ on discoid diabetic platelets and that platelet aggregates can be eliminated by PI 3-kinase inhibition, but not by anti-thrombotics aspirin or clopidogrel.

## Introduction

Diabetes mellitus is becoming one of the major threats to human health and longevity in the 21st century. Based on current trends, children born after the year 2000 will have up to a 30% life-time risk of developing diabetes, leading to a 20–30% reduction in life expectancy^1^. Most individuals with diabetes die from the complications of cardiovascular diseases, particularly the acute coronary syndromes. Individuals with diabetes develop more widespread and advanced atherosclerotic lesions, and these plaques are more prone to rupture compared to non-diabetic individuals. Moreover, the thrombotic response at sites of atherosclerotic plaque rupture is typically exaggerated in diabetes, increasing the risk of vaso-occlusive thrombus formation, myocardial infarction and sudden death.

Platelets play a central role in the development of coronary disease by initiating and propagating plaque development, as well as promoting thrombus formation on the surface of disrupted plaques^2^. Platelets from individuals with diabetes are more reactive than platelets from non-diabetics, as evidenced by an increased response to soluble agonist stimulation^3–5^ along with enhanced adhesion and aggregation responses on thrombogenic surfaces^6,7^. They are also more effective at supporting blood coagulation and thrombin generation^8^. The mechanisms regulating platelet reactivity in diabetic patients are complex and not completely understood. Following stimulation, platelets from diabetic patients have elevated levels of cytosolic calcium^9^ and generate higher levels of thromboxane A2 (TxA_2_)^10,11^. Chronic hyperglycemia leads to non-enzymatic glycation of platelet membrane proteins^12,13^ and upregulation in the function of the platelet P_2_Y_12_ receptor^14,15^. Reduced intracellular levels of antioxidants^16^, enhanced formation of soluble advanced glycation end products (AGEs)^17^, oxidative inactivation of the SERCA2 Ca-ATPase^18^, as well as mitochondrial dysfunction contribute to alterations in platelet reactivity in diabetics^19,20^.

The clinical management of thrombosis risk for individuals with diabetes is complicated by the fact that platelets from diabetics are less responsive to the platelet inhibitory effects of the conventional antiplatelet agents, aspirin, and clopidogrel^21^. Despite the introduction of more potent P_2_Y_12_ antagonists, such as ticagrelor, diabetes remains associated with an elevated incidence of thromboembolic complications. Interestingly, integrin α_IIb_β_3_ antagonists, the most potent class of antiplatelet agents, appear to work most effectively in diabetics^22,23^, indicating that dysregulation of integrin α_IIb_β_3_ function is likely to be an important process underlying the diabetic prothrombotic phenotype.

The manner in which diabetes effects α_IIb_β_3_ activation and the kinetics of thrombus growth remains ill-defined^24^. This is likely to be clinically important as diabetics are more likely to form stable vaso-occlusive thrombi that precipitate organ injury^25^. Experimental studies have demonstrated that the efficiency of thrombus growth in vivo is influenced by the interplay of two distinct, but complementary, platelet aggregation mechanisms^26^. The first involves a rheology-dependent (biomechanical) platelet aggregation mechanism that is primarily mediated by discoid platelets. This mechanism is important for the initial recruitment of platelets to sites of vascular injury, particularly under conditions of disturbed blood flow^27^. The second is a soluble agonist-dependent aggregation mechanism that stabilizes formed aggregates. The biomechanical platelet aggregation mechanism primarily involves discoid platelets in a low-activation state. Aggregation of these platelets is initiated by hemodynamic shear gradients and requires the co-operative adhesive function of the platelet receptors GPIb and integrin α_IIb_β_3_^27^. The second aggregation mechanism involves agonist-induced platelet activation that primarily serves to upregulate integrin α_IIb_β_3_ adhesive function and stabilize platelet aggregates. As a consequence, developing thrombi exhibit a heterogeneous structure of platelets in various degrees of activation and stability, ranging from fully activated and degranulated platelets in the stable thrombus ‘core’, to minimally activated, weakly adherent discoid platelets in the dynamic thrombus outer ‘shell’^26–28^.

In this report, we have examined the impact of chronic hyperglycemia on platelet responses to biomechanical and agonist stimulation using a streptozotocin (STZ) murine model of diabetes. Surprisingly, chronic hyperglycemia for up to 10 weeks in the mouse, did not result in increased platelet sensitivity to soluble agonist stimulation in vitro and in vivo. In contrast, chronic hyperglycemia resulted in an enhancement in biomechanical α_IIb_β_3_ activation, leading to a shear and red blood cell (RBC)-dependent increase in discoid platelet adhesion and aggregation in vitro and in vivo. Biomembrane force probe (BFP) analysis of platelet adhesion receptor–ligand kinetics at a single-platelet level indicates that in diabetics, increased sensitivity of platelets to shear forces is due in part to dysregulated integrin α_IIb_β_3_ compression force sensing.

## Results

### Diabetes enhances discoid platelet aggregation in vivo

**Fig. 1:**
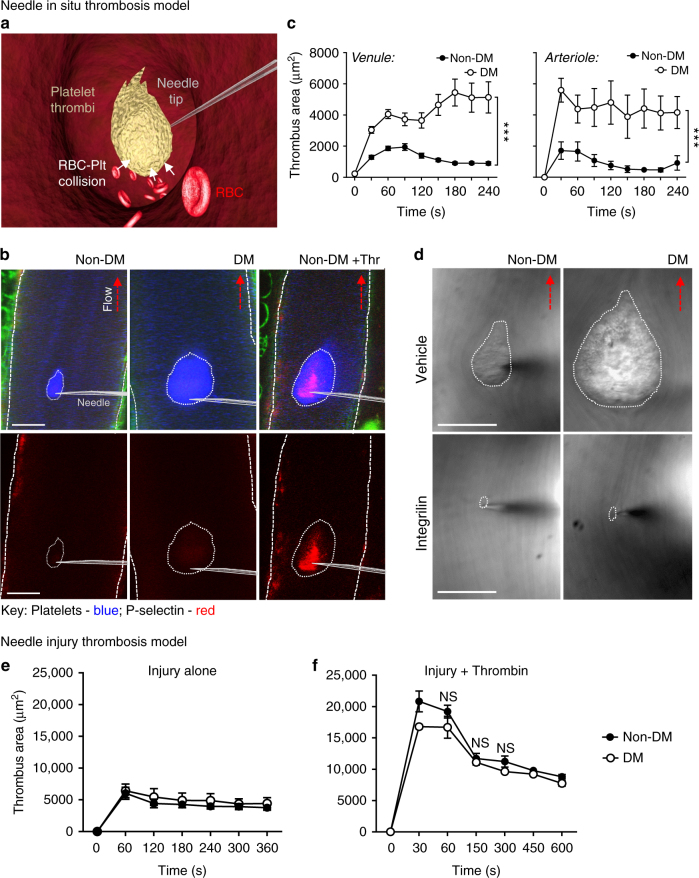
Diabetes exaggerates discoid platelet thrombus growth under local flow disturbance in vivo. Control non-diabetic (non-DM) and diabetic (DM) mice were administered Dylight 649-anti-GPIbβ Ab and Alexa 546-anti-P-selectin Ab, prior to subjection to the ‘needle in situ’ model of thrombosis, as described under ‘Methods section’. In some experiments (**d**), mice were treated with vehicle or integrilin (as described) prior to needle injury. **a** Schematic illustration of the ‘needle in situ’ thrombosis model, wherein a microinjector needle is inserted into the center of the vessel lumen to create local flow disturbance. Thrombus formation is initiated at the needle tip and propagated by aggregating discoid platelets. Red blood cells (RBCs) have a collisional effect on platelets within the growing thrombus. **b** Thrombus growth (blue) and P-selectin expression (red) were monitored in real-time via confocal microscopy, with representative images depicting P-selectin^−^^ve^ thrombi (blue) in both non-DM and DM mice, 4 min post-needle insertion. P-selectin^+ve^ thrombi were detected only following injection of thrombin (green: collagen autofluorescence). **c**, **d** Thrombus surface area of non-DM and DM mice was quantified at the indicated time points post-needle insertion. **c** Results are expressed as mean ± s.e.m., *n* = 3 mice (6–8 thrombi per mouse examined in venules and 5 thrombi per mouse in arterioles). **d** Representative DIC images depicting thrombi (dotted line) forming 4 min post-needle insertion in non-DM and DM mice, either untreated (control) or treated with α_IIb_β_3_ antagonist integrilin (4 mg kg^−1^, i.v.). **e**, **f** In some experiments, thrombi induced in non-DM and DM mice by needle perturbing mesenteric venules alone (**e**) was compared to local injection of thrombin (100 U mL^−1^) (**f**). Thrombus formation was monitored using DIC intravital microscopy and thrombus surface area quantified at the indicated injury times. Results (**e**, **f**) are expressed as mean ± s.e.m. of *n* = 3 mice, (2–3 thrombi per mouse). For all studies, results were assessed using an unpaired, two-tailed Student’s *t*-test, where *p < 0.5; ****p < 0.0001. Scale bars = 50 μm

We initially investigated the impact of hyperglycemia on platelet adhesive function in vivo, using the streptozotocin (STZ)-induced mouse model of type 1 diabetes^29,30^. This model allows precise control over the onset of hyperglycemia and enables assessment of platelet function independent of the confounding effects of other cardiovascular risk factors or overt vascular disease^30,31^. Hyperglycemia (blood glucose levels of >15 mM) was confirmed 1 week post streptozotocin injection and in vivo thrombosis studies were performed on mice 7–10 weeks post streptozotocin injection. In initial studies, we investigated the impact of disturbed blood flow on the platelet aggregation response in diabetic mice using a needle in situ model of platelet thrombus formation^32,33^. In this model, the tip of a microinjector needle is inserted into the center of the lumen of a mesenteric vessel, creating a local region of flow disturbance (Fig. 1a). Discoid platelet aggregate formation was limited to the needle tip and did not occur at the site of endothelial needle injury, allowing examination of platelet responses independent of changes in the vessel wall. This feature provides a significant advantage, given that endothelial dysregulation in diabetes can promote platelet-vessel wall interactions. Notably, >95% of the platelets recruited in the needle in situ model displayed undetectable P-selectin surface expression by immunofluorescence staining, confirming that these platelets were in a low-activation state (Fig. 1b). Platelet aggregates forming in non-diabetic mice were dynamic and highly unstable (Supplementary Movie 1). Initial accrual of platelets to the needle tip occurred in a time-dependent fashion (Fig. 1c, 0–120 s) followed by subsequent loss of platelets from the outer shell, leading to a reduction in surface area over time (Fig. 1c, 120–240 s). Strikingly, in diabetic mice, both the rate and extent of discoid platelet aggregation were markedly increased compared to non-diabetic controls (Fig. 1b–d). These platelets remained in a low-activation state as they failed to express surface P-selectin (Fig. 1b). Analysis of the surface area of thrombi formed in diabetic mice revealed a 3- to 4-fold increase over a 4-min timeframe (Fig. 1c). A similar enhanced thrombotic response around the needle tip was also observed in the arterial circulation of diabetic mice (Fig. 1c). In control studies, we confirmed that discoid platelet aggregation was integrin α_IIb_β_3_-dependent, as pretreating mice with the integrin α_IIb_β_3_ antagonist, integrilin (4 mg kg^−1^), completely inhibited discoid platelet aggregation around the needle tip in both diabetic and non-diabetic mice (Fig. 1d and Supplementary Fig. 1).

Discoid platelet aggregates are stabilized by the generation of soluble agonists at sites of vascular injury^26,27^. Human diabetic platelets have enhanced responsiveness to soluble agonist stimulation, manifesting as increased rates of platelet aggregation at threshold levels of platelet stimulation^34,35^. To investigate whether mouse diabetic platelets had increased responsiveness to soluble agonist stimulation in vivo, we utilized an endothelial needle injury model of thrombosis, which perturbs, but does not denude, the endothelium at the site of injury and enables the local microinjection of soluble agonists at sites of thrombus formation^36^. Platelet activation in this model, prior to the injection of soluble agonists, is critically dependent on the generation of thrombin on the perturbed endothelium^36,37^. Interestingly, the initial recruitment and formation of platelet aggregates in this model were no different between control and diabetic mice (Fig. 1e). Moreover, when platelet activation was amplified by microinjecting thrombin (Fig. 1f) or ADP (Supplementary Fig. 2), there was no difference in the rate, extent or stability of thrombus growth between control and diabetic mice. Overall, these findings suggest that 7–10 weeks of hyperglycemia in the mouse has a major impact on discoid platelet aggregation, yet surprisingly, has limited impact on platelet responsiveness to soluble agonist stimulation in vivo.

### Diabetes enhances α_IIb_β_3_ dependent platelet adhesion in vivo

**Fig. 2:**
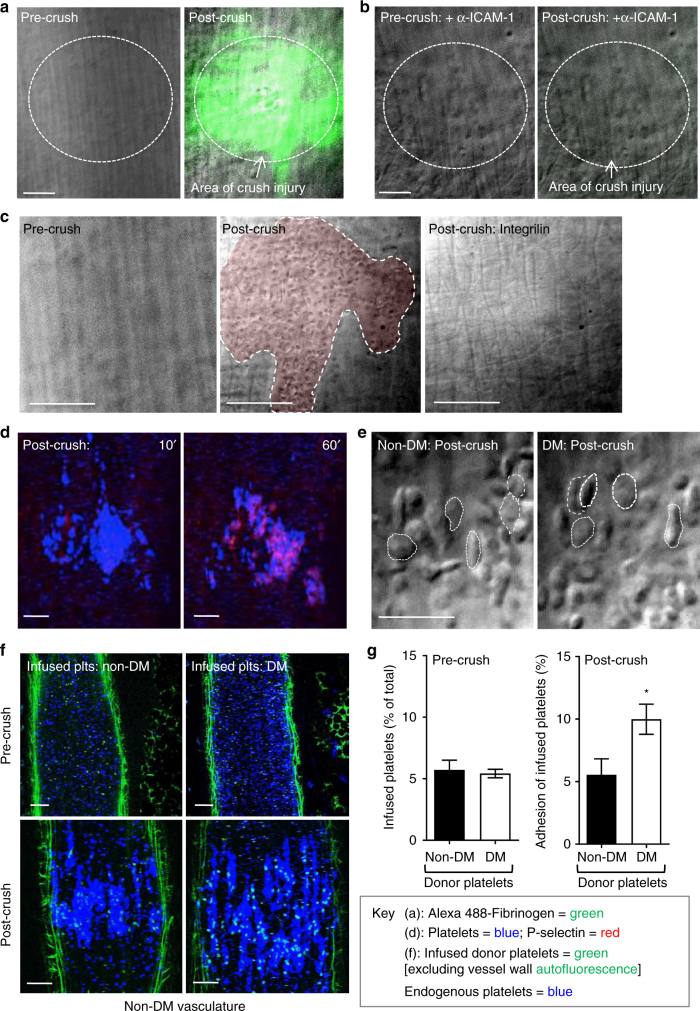
Enhanced diabetic platelet-endothelial interactions in vivo. The mesenteric venules of non-DM or DM mice were subjected to crush injury using a blunt needle, as detailed under ‘Methods section’. **a**–**c** C57Bl/6 mice were administered Alexa 488-fibrinogen alone (**a**), or together with an anti-ICAM-1 blocking Ab (Clone YN1, 200 μg per mouse) (**b**), prior to injury. Representative DIC/fluorescence overlay images demonstrate ICAM-1-dependent fibrinogen deposition (green) on the endothelium, post- but not pre-crush injury (broken circles). Scale bars = 25 μm. In some experiments, mice were pre-administered integrilin (4 mg kg^−1^) (**c**), prior to injury, with representative DIC images demonstrating platelet adhesion to the crushed endothelium (shaded in red) in vehicle treated- (middle panel—post-crush), but not integrilin-treated mice (right panel—Post-crush: integrilin), or pre-crush injury (left panel). Scale bars = 25 μm. **d** C57BL/6 mice were administered Alexa 546-anti-P-selectin Ab and DyLight 649-anti-GPIbβ Ab, prior to crush injury. Representative confocal images depict the absence P-selectin (red) expression early after injury (10 min), instead developing at 60 min post-crush injury on endothelial adherent platelets (blue). Scale bars = 50 μm. **e** Representative DIC images depict the majority of the endothelial adherent platelets in non-diabetic (non-DM) and diabetic (DM) mice 10 min post crush injury (some discoid platelets marked with dotted circles) retain a discoid morphology. Scale bars = 10 μm. **f**, **g** Non-DM and DM platelets were isolated, labeled with an Alexa 488-anti-GPIbβ Ab (100 μg kg^−1^), and then infused into recipient non-DM mice, pre-administered DyLight 649-anti-GPIbβ antibody (100 μg kg^−1^), at a donor/recipient ratio of 1:10. **f** Representative confocal images demonstrating both infused (green) and endogenous (blue) platelets in mesenteric circulation of recipient non-DM mice, pre- (upper panel) and post- (lower panel) crush injury. Scale bars = 50 μm. **g** The adherent donor and recipient platelets to the crushed endothelial area was quantitated by determining their fluorescence intensities, and expressed as a percentage of the recipient endogenous platelets within the same crushed surface area (green: collagen autofluorescence). All results represent the mean ± s.e.m. of *n* = 4 mice (six injuries per mouse), assessed by unpaired, two-tailed Student’s *t*-test, where *p < 0.5

In addition to mediating platelet aggregation, integrin α_IIb_β_3_ also promotes platelet-endothelial adhesive interactions in vivo^38^. This is mediated by the ICAM-1-dependent deposition of fibrinogen on the endothelial cell surface, which provides a reactive surface for platelet recruitment via integrin α_IIb_β_3_^38,39^. To investigate the impact of diabetes on platelet-endothelial adhesion, we established a mechanical endothelial injury model using micromanipulators and blunted microinjector needles (see Methods section and Supplementary Fig. 3a) that leads to the localized deposition of fibrinogen onto the surface of endothelial cells (Fig. 2a), which is dependent upon endothelial ICAM-1 binding (Fig. 2b). Subsequent platelet recruitment occurred exclusively at sites of fibrinogen deposition (Fig. 2c and Supplementary Fig. 3b), and was integrin α_IIb_β_3_ dependent, since it was completely inhibited by pretreating mice with integrilin (Fig. 2c). Most adherent platelets expressed undetectable levels of P-selectin (Fig. 2d) and retained a discoid morphology (Fig. 2e) in the first 10 min of adhesion, suggesting that most cells remained in a low-activation state. Platelets also readily adhered to sites of endothelial injury in STZ-treated mice and retained their discoid morphology; however, quantification of platelet numbers was difficult due to the rapid, confluent adhesion response and the propensity of diabetic platelets to form aggregates. To overcome this problem, we performed adoptive transfer experiments in which diabetic platelets were infused into a non-diabetic recipient mouse that underwent mechanical endothelial injury, enabling examination of diabetic platelet adhesive function in the context of a non-diabetic perturbed endothelium. In these studies, diabetic platelets were isolated, labeled with Alexa 488-anti-GPIbβ antibody (Ab), then infused into non-diabetic control mice at a ratio of 1/10 (infused/endogenous) with endogenous platelets labeled with DyLight 649-anti GPIbβ Ab (Methods section). Prior to injury, fluorescence imaging confirmed that the ratio of infused/endogenous platelets was comparable between the non-diabetic and diabetic platelet infusions (Fig. 2f, g). Following mechanical endothelial injury, both donor and endogenous platelets were deposited on the injured endothelium. Analysis of the ratio of donor vs. endogenous platelets revealed a twofold increase in diabetic donor platelet adhesion over the non-diabetic donor platelets (Fig. 2f, g), confirming that the enhanced platelet-endothelial interaction in diabetic mice is related to alterations in diabetic platelet adhesive function. These studies provide evidence that diabetes enhances the integrin α_IIb_β_3_-dependent adhesive function of platelets in vivo.

### Diabetes modulates shear selective α_IIb_β_3_ adhesive function

**Fig. 3:**
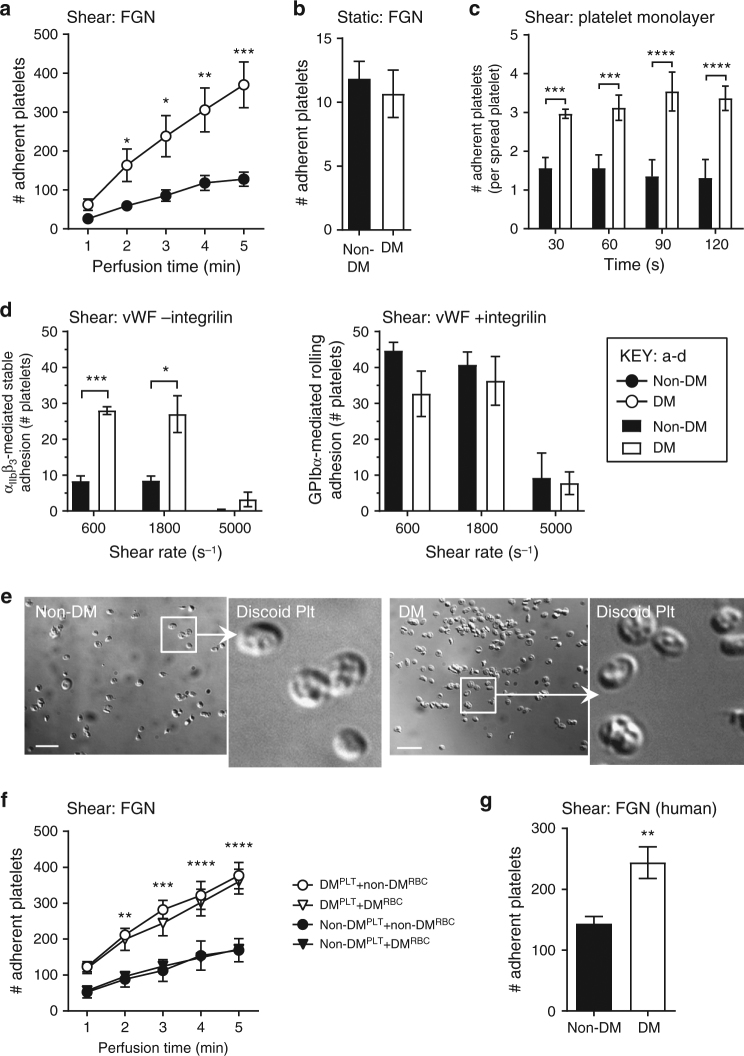
Diabetes enhances integrin α_IIb_β_3_ mediated platelet adhesion under shear. **a** Hirudinated whole blood from non-DM or DM mice were perfused through fibrinogen (FGN) matrices for 5 min at 600 s^−1^, and the number of adherent platelets determined at the indicated perfusion times. **b** Isolated mouse platelets were allowed to adhere to FGN matrices under static conditions for 5 min, and the number of adherent platelets quantitated. **c** Hirudinated whole blood from non-DM and DM mice were pretreated with amplification loop blockers (ALBs): indomethacin (10 μM), MRS2179 (100 μM), and 2-MeSAMP (10 μM), then perfused over spread platelets at 1800 s^−1^. The number of adherent platelets per spread platelet was quantified at 30 s intervals for 120 s. **d** Hirudinated mouse whole blood was perfused over mouse vWF matrices at 600, 1800, and 5000 s^−1^, as detailed under Methods section. The numbers of stationary (−integrilin) vs. transient/rolling (+integrilin) platelets were determined at 1 min perfusion time. **e** Hirudinated mouse whole blood was perfused through FGN matrices at 600 s^−1^ for 1 min, and adherent platelets were fixed immediately. Representative DIC images depict the discoid morphology of the adherent non-DM and DM mouse platelets. Scale bars = 5 μm. **f** Platelets isolated from non-DM and DM mice were reconstituted with isolated red blood cells (RBCs) from the same (autologous) or counterpart (heterologous) mice, prior to perfusion over matrices at 600 s^−1^. The number of adherent non-DM and DM platelets was determined at the indicated perfusion times. **g** Hirudinated whole blood from non-DM or DM humans was perfused through FGN matrices for 5 min at 300 s^−1^, and the number of adherent platelets determined at 5 min perfusion time. Results performed with human samples are expressed as mean ± s.e.m. of *n* = 16 for non-DM and *n* = 18 for DM patients. All other results represent *n* = 3 mice, with each perfusion performed in duplicates or triplicates. NS = not significant, *p* ≥ 0.05; *p < 0.05; **p < 0.01; ***p < 0.001; ****p < 0.0001, assessed by unpaired, two-tailed Student’s *t*-test

To examine whether the shear–dependent interaction between platelet integrin α_IIb_β_3_ and immobilized fibrinogen (FGN) was dysregulated by diabetes, whole blood was perfused over FGN matrices at both venous (600 s^−1^) and arterial (1200 s^−1^) shear rates. The number of diabetic platelets adherent to FGN was increased 2–3-fold relative to non-diabetic platelets (Fig. 3a and Supplementary Fig. 4), recapitulating our in vivo findings. Interestingly, this enhancement was shear-specific as there was no increase in diabetic platelet adhesion to immobilized FGN under static conditions (Fig. 3b). A similar shear-dependent increase in discoid platelet adhesion was also observed when blood was perfused over spread platelet monolayers at 1800 s^−1^ in the presence of inhibitors of ADP and TxA_2_ to prevent paracrine activation of tethering discoid platelets (Fig. 3c). These results indicate that diabetic platelets display enhanced adhesion under the both venous and arterial shear conditions (Fig. 3c).

The adhesion of discoid platelets to the spread platelets under high shear is dependent on the adhesion receptors GPIb and integrin α_IIb_β_3_^40^. To further define the receptors responsible for the enhanced platelet adhesion in diabetes, we perfused whole blood over vWF matrices at 600, 1800, and 5000 s^−1^. Significantly, the number of α_IIb_β_3_ mediated stationary platelets was 2–3-fold higher than non-diabetic controls at all shear rates (Fig. 3d). In contrast, GPIb mediated platelet rolling was not significantly different (Fig. 3d). These data demonstrate that shear-enhanced adhesion of diabetic platelets is selective to integrin α_IIb_β_3_, irrespective of its ligands, and occurs at both venous and arterial shear rates.

Analysis of the morphology of platelets stably adherent to fibrinogen confirmed that both non-diabetic and diabetic platelets retained their discoid morphology during the early stages of perfusion (Fig. 3e). Moreover, the enhanced adhesive response of discoid platelets seen in whole blood was recapitulated when washed diabetic platelets were reconstituted with either autologous diabetic RBCs or heterologous non-diabetic RBCs (without plasma) (Fig. 3f). This indicates that the hyperadhesive phenotype of diabetic platelets is intrinsic to platelets, rather than secondary to activating stimuli in plasma or due to the effects of diabetic RBCs^41^. Importantly, a similar enhancement in integrin α_IIb_β_3_ mediated platelet adhesion to immobilized fibrinogen was also observed in human diabetic platelets. Perfusion of anticoagulated whole blood from patients with type 1 diabetes (*n* = 18, age range 20–64; mean age 47 years) over an immobilized fibrinogen matrix resulted in a twofold increase in platelet adhesion relative to non-diabetic healthy donors (Fig. 3g). These latter findings demonstrate that the shear-dependent increase in platelet-fibrinogen interactions is not species-specific, occurring in both human and mouse diabetic platelets.

To further investigate the possibility of a shear-selective increase in integrin α_IIb_β_3_ adhesive function in diabetic platelets we examined soluble agonist stimulated integrin α_IIb_β_3_ activation and platelet aggregation. In control studies, we confirmed that there were no significant differences in the basal levels of integrin α_IIb_β_3_ expression and affinity in control and diabetic mouse platelets (Supplementary Fig. 5a, b). Moreover, soluble agonist-induced integrin α_IIb_β_3_ activation and platelet aggregation were similar between control and diabetic mouse platelets (Table 1 and Supplementary Fig. 5a,b). This was apparent with both the weak platelet agonist ADP and threshold concentrations of the more potent platelet agonist PAR4 activating peptide (PAR4-AP). Moreover, these platelet responses were identical from mice with either acute (1-week post STZ) or chronic hyperglycemia (7 weeks post STZ) (Supplementary Fig. 5b). Similar findings were apparent with platelet degranulation, in which α-granule exocytosis, as measured by P-selectin expression, was not significantly different between non-diabetic and diabetic platelets in response to PAR4-AP in either whole blood or isolated washed platelet assays (Table 1 and Supplementary Fig. 5c). These studies confirm our in vivo findings, that persistent hyperglycemia for up to 10 weeks in the mouse does not markedly enhance agonist-induced platelet activation. Rather, they indicate that chronic hyperglycemia preferentially enhances the adhesive function of integrin α_IIb_β_3_ under conditions of hemodynamic shear stress.

**Table 1:** Diabetic platelets do not express increased basal levels of α_IIb_β_3_ or display enhanced responses to soluble agonist stimulation

	Agonist (µM)	Non-DM	DM	Significance
Integrin α_IIb_β_3_ expression	Untreated	179.8 ± 4.52	203.3 ± 30.9	NS
	Untreated (human)	394.5 ± 24.6	488.2 ± 49.6	NS
Integrin α_IIb_β_3_ activation (JON/A binding)	Untreated	5.67 ± 0.88	4.67 ± 0.67	NS
	ADP (1)	26.98 ± 1.21	27.57 ± 2.94	NS
	ADP (10)	111.2 ± 15.8	102.7 ± 19.6	NS
	PAR-4 AP (100)	14.67 ± 1.20	24.33 ± 5.23	NS
	PAR-4 AP (300)	82.33 ± 3.71	71.33 ± 1.76	NS
P-selectin expression	PAR-4 AP (100)	27.57 ± 2.90	30.11 ± 4.82	NS
	PAR-4 AP (300)	50.48 ± 2.34	46.88 ± 4.69	NS
P-selectin expression (isolated platelets)	PAR-4 AP (100)	23.01 ± 2.17	28.22 ± 4.92	NS
	PAR-4 AP (300)	54.36 ± 5.55	49.19 ± 5.42	NS
Aggregation (% maximal)	ADP (1)	42.4 ± 1.4	41.6 ± 3.4	NS
	ADP (10)	59.3 ± 2.9	57.0 ± 10.0	NS

To assess integrin α_IIb_β_3_ expression (Leo.F2 binding for mouse and HIP8 binding for human), activation (JON/A binding) and P-selectin expression in non-DM and DM platelets (mouse, if not stated otherwise), hirudinated whole blood (1/20 diluted, if not stated otherwise) or isolated washed platelets were stimulated with the indicated concentrations of ADP or PAR-4 activating peptide (PAR4-AP) for 10 min, in the presence of PE-JON/A Ab or FITC-anti-P-selectin Ab, respectively, then subjected to flow cytometry. Results are expressed as the geometric mean of fluorescence intensity. For platelet aggregation, platelet rich plasma from non-DM and DM mice were subjected to aggregometry using 1 or 10 µM ADP. Results are expressed as maximal extent of platelet aggregation (%). All results represent mean ± s.e.m. of *n* ≥ 3 independent mouse experiments. For human results, *n* = 16 for non-DMs and *n* = 18 for DMs. NS = not significant, *p* ≥ 0.05. assessed using an unpaired, two-tailed Student’s *t*-test.

### Compression forces enhance α_IIb_β_3_ ligand interactions

**Fig. 4:**
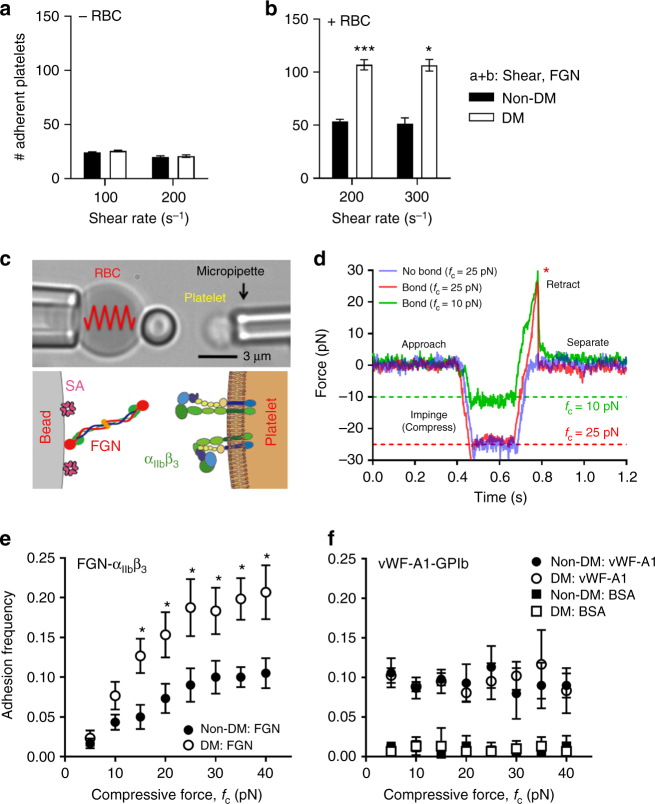
Compressive force dependent enhancement in integrin α_IIb_β_3_–fibrinogen interaction in diabetic platelets. **a**, **b** Isolated platelets from non-DM and DM mice were perfused over FGN matrices at the indicated shear rates, in the absence (**a**, −RBC) or presence (**b**, +RBC) of RBC reconstitution. The number of adherent platelets was determined following 3 min of perfusion time. **c** Schematic illustration of the biomembrane force probe (BFP) setup. (Top) The micropipette-aspirated platelet is driven forward to touch and then retracted back from the adhesive ligand- (i.e., FGN or vWF) coated bead, which is attached to a RBC aspirated by another micropipette. (Bottom): each ‘touch-retract’ cycle allows platelet surface integrin α_IIb_β_3_ to associate and then dissociate with FGN on the bead. **d** Illustration of bond detection using BFP. The platelet is driven to touch the probe bead under a compressive force (*f*_c_) of 10 (green) or 25 pN (red), held in contact for 0.2 s, and then retracted. In a ‘bond’ event (green and red), a loading force increases until the bond rupture (red asterisk), whereas a ‘no-bond’ event shows zeroforce (blue). **e**,** f** Non-DM (closed circles) and DM (open circles) mouse platelets were subjected to 50 BFP cycles under the indicated compressive forces (*f*_c_ = 5–40 pN) using beads coated with FGN (**e**), vWF-A1 or BSA (**f**), and the number of ‘bond’ events over the total number of events was analysed and expressed as adhesion frequency. All results are expressed as mean ± s.e.m. of *n* = 3 mice. For each experiment, adhesion frequencies were measured and averaged from ≥4 platelet**–**bead pairs. Results were assessed using an unpaired, two-tailed Student’s *t*-test, where *p < 0.5; **p < 0.01

In our washed platelet and red blood cell (RBC) reconstitution assays we made the surprising observation that the shear-dependent increase in diabetic platelet adhesion was RBC-dependent. Perfusion of isolated mouse platelets over FGN in the absence of RBCs yielded comparable numbers of adherent diabetic and non-diabetic platelets (Fig. 4a). After reconstituting the diabetic mouse platelets with non-diabetic human RBCs, the hyperadhesive phenotype was rescued (Fig. 4b), demonstrating a critical role for RBCs in promoting the diabetic hyperadhesive phenotype. RBCs have a well-defined role in enhancing platelet transport to the vessel wall (margination)^42,43^ and recent rheological and modeling studies demonstrated that RBCs push and subject platelets to collision forces (compression)^44,45^, although how compression forces influence platelet adhesive function remains largely unknown. To address this, we developed a novel compression assay using a biomembrane force probe (BFP). The probe bead was functionalized with FGN (Fig. 4c) and driven to repetitively touch a discoid platelet aspirated by another micropipette with a controlled compressive force (*f*_c_). If a bond connects the bead and platelet during their separation, the RBC would be stretched and report a tensile force (Fig. 4d). The intermittent “touch and retract” cycles mimic-specific aspects of the platelet ‘stop-and-go’ adhesive behavior under flow^46,47^. The adhesion frequencies (*P*_a_ = the number of productive touches divided by the number of 50 total touches) reflect the likelihood to find a receptor–ligand bond or bonds at the time of detection. To examine RBC-platelet collision effect, we measured the platelet adhesion frequencies with various compressive forces ranging from 5 to 40 pN. Interestingly, the adhesion frequency *P*_a_ was found to increase with the compressive force *f*_c_ (Fig. 4e). This effect was integrin-specific, since replacing FGN with BSA on the BFP eliminated the adhesion frequencies at all forces tested (Fig. 4f). Moreover, vWF-A1 binding to GPIb was not compressive force dependent throughout the force ranges tested (Fig. 4f), suggesting that the increased integrin adhesion frequency with compressive force cannot be explained solely by an increase in the bead-platelet contact area^48^.

Notably, platelets from diabetics were more responsive to compressive force than from non-diabetics, manifesting as a differential adhesion frequency that commenced at a threshold compressive force of 15 pN, progressively increasing with *f*_c_, and reaching a maximum (twofold increase) at saturating forces of *f*_c_ > 20 pN (Fig. 4e). Again, replacing the FGN with BSA on the BFP eliminated the adhesion frequencies at all forces tested (Fig. 4f). Moreover, the enhancing effects of diabetes on compression force sensing appeared α_IIb_β_3_-selective, as there was no difference in vWF-A1–GPIb-specific binding at any compressive force applied to control or diabetic platelets (Fig. 4f). Thus, the integrin-mediated diabetic hyperadhesive phenotype observed in microslide perfusion experiments can be recapitulated using the BFP adhesion frequency assay, while also allowing precise control of the compressive force. This suggests that amplifying α_IIb_β_3_ integrin-dependent adhesion in response to compressive force represents a mechanism for diabetes to enhance platelet adhesion.

### Compressive forces enhance α_IIb_β_3_ on rate in diabetic platelets

**Fig. 5:**
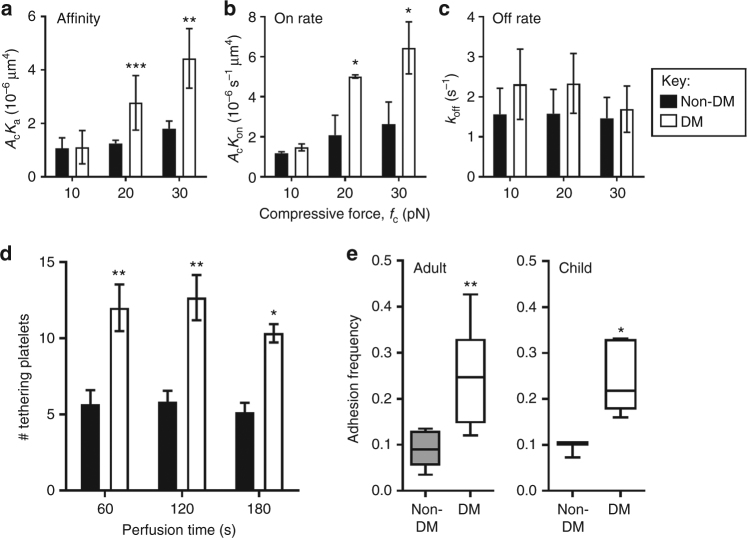
Compressive forces enhance integrin α_IIb_β_3_–fibrinogen bond formation in diabetic platelets. **a**–**c** Platelets from non-DM and DM mice were subjected to BFP cycles using FGN-coated beads at various contact times to determine the 2D affinity (**a**), on-rate (**b**) and off-rate (**c**) of integrin α_IIb_β_3_ at *f*_c_ = 10, 20 and 30 pN, as detailed under Methods section. **d** Hirudinated whole blood from non-DM or DM mice were perfused over FGN matrices at 600 s^−1^, and the number of tethered platelets at the indicated perfusion times was quantified. Results are expressed as mean ± s.e.m. of *n* = 3–4 mice. **e** Human platelets from non-DM and DM adults or children were subjected to 50 BFP cycles using beads coated with FGN at *f*_c_ = 20 pN, and adhesion frequency determined. Data are represented in box and whisker plot format, with median, first and third quartiles outlined by the box, and minimum and maximum values of the data set denoted by whiskers (*n* = 7 for both non-DM and DM adults; *n* = 3 for non-DM children; *n* = 5 for DM children) *p < 0.5; **p < 0.01, assessed by unpaired, two-tailed Student’s *t*-test

To identify which biophysical properties of the molecular interaction of diabetic integrin α_IIb_β_3_ are impacted by compressive force, we measured the 2D kinetics of the FGN–α_IIb_β_3_ integrin interaction on both control and diabetic mouse platelets at compression forces *f*_c_ = 10, 20, and 30 pN. Strikingly, integrin α_IIb_β_3_ on diabetic platelets displayed a significantly enhanced adhesion frequency at 20 and 30 pN, but not 10 pN, compared with integrin α_IIb_β_3_ on control platelets. The adhesion frequencies (*P*_a_) were measured over a range of contact times (*t*_c_) and various compression forces (Supplementary Fig. 6). Fitting the *P*_a_ vs. *t*_c_ curves with the single-step monomeric bimolecular interaction kinetics model returned the zero-force effective 2D kinetic on-/off-rates and affinity^47,49^. The BFP kinetic analysis revealed that diabetic platelets displayed a comparable 2D affinity to control platelets at *f*_c_ = 10 pN; however, a 2–3-fold increase at 20 and 30 pN (Fig. 5a). Notably, this increase is mainly contributed by the on-rate (Fig. 5b) as the off-rate was not significantly different between non-diabetic and diabetic platelets (Fig. 5c). These kinetic analyses were consistent with the increased tethering of mouse diabetic platelets observed during perfusion of diabetic whole blood over a fibrinogen matrix (Fig. 5d). In order to confirm that these findings were not specific to the mouse model of diabetes, and further applied to all stages of diabetes, platelets from patients with type 1 diabetes (*n* = 7, age range 20–71; mean age = 35 yrs) were isolated and the adhesion frequency examined in an identical fashion using the BFP assay (*f*_c_ = 20 pN). In accordance with results obtained from mouse platelets, FGN binding was enhanced in diabetic human platelets (Fig. 5e, left). Moreover, we also investigated a group of pediatric patients with type 1 diabetes (*n* = 5, age range 7–18; mean age = 13 yrs) with more recent onset of diabetes and therefore less likelihood of overt vascular disease. Similar to adults with type 1 diabetes, this pediatric group also exhibited enhanced platelet adhesion frequency to FGN by at *f*_c_ = 20 pN (Fig. 5e, right). These findings indicate that diabetes increases the compressive force sensitivity of platelets, thereby enhancing the kinetic association rates of FGN–α_IIb_β_3_ integrin bonds and accelerating platelet recruitment under flow.

### Compression force dependent activation of integrin α_IIb_β_3_

**Fig. 6:**
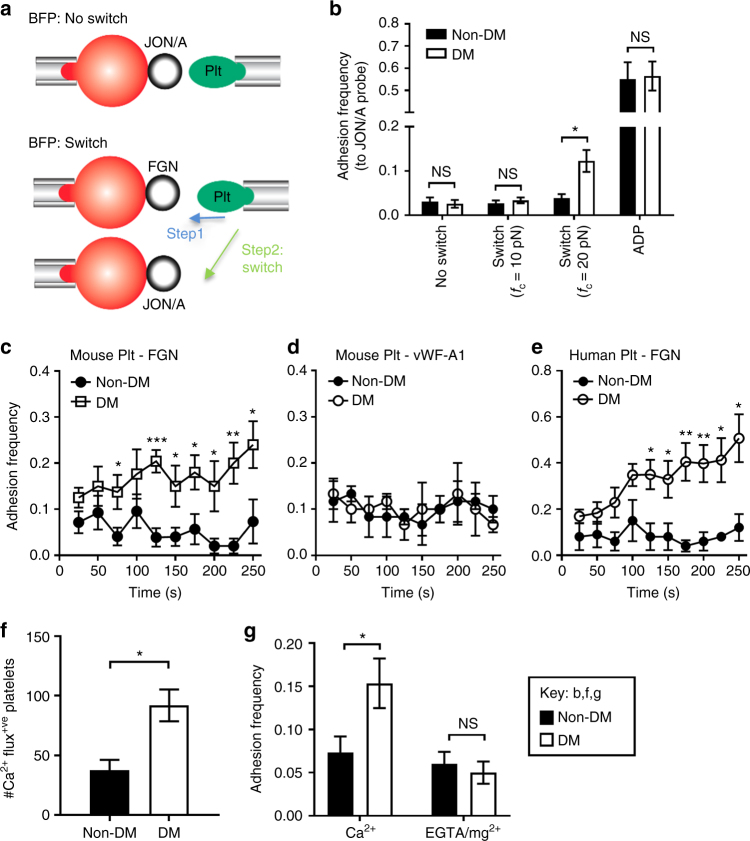
Compression force-dependent activation of integrin αII_b_β_3_. **a** Schematics of ‘no switch’ BFP (top) and ‘switch’ dual BFP setup (bottom). In the ‘switch’ setup, the platelet is first subjected to 50 cycles with a FGN coated bead at a compressive force of 20 pN (step 1), and then switched to 50 cycles with bead coated with JON/A. **b** Mouse platelet adhesion frequencies with JON/A were determined under conditions of no switch, or switch at the indicated compressive forces (*f*_c_ = 10, 20 pN), or ADP stimulation (10 µM). **c**–**e** Non-DM and DM platelets from mice or humans were subjected to 100 BFP cycles at *f*_c_ = 20 pN using FGN (**c**, **e**) or vWF-A1 (**d**) beads. Adhesion frequency was analyzed for every 10 consecutive cycles and plotted over time (2.5 s/cycle). **f** Isolated mouse non-DM and DM platelets were loaded with calcium dyes Oregon Green BAPTA and Fura Red, reconstituted with RBCs from the same mouse, and perfused over FGN matrices at 600 s^−1^. The calcium changes in individual adherent platelets were monitored and the number of adherent platelets displaying Ca^2+^ flux analysed. **g** Non-DM and DM mouse platelets were resuspended in Tyrode’s buffer containing 1 mM Ca^2+^ or 1 mM EGTA/Mg^2+^, subjected to 50 BFP cycles using FGN-beads at *f*_c_ = 20 pN, and adhesion frequency determined. All results are expressed as the mean ± s.e.m. of *n* = 3 mice and assessed by unpaired, two-tailed Student’s *t*-test, where *p < 0.5; **p < 0.01

We next asked whether the α_IIb_β_3_ molecule was differentially activated on diabetic discoid platelets in response to compressive force to adopt an active conformation. Importantly no significant differences in the active conformation of α_IIb_β_3_ on non-diabetic and diabetic platelets were observed by JON/A binding (a mAb specific to the active conformation of integrin α_IIb_β_3_) in the absence of force using flow cytometry^50^ (Table 1 and Supplementary Fig. 5a,b). Therefore, we combined the compression assay and a newly developed “switch assay” using a dual BFP setup^51^ (Fig. 6a lower). The aspirated platelet was first driven to touch 50 cycles with the FGN-bearing probe under a constant compressive force, and then switched to the second probe coated with JON/A (Fig. 6a). In sharp contrast to the force-free flow cytometry assay (Supplementary Fig. 5a,b), the above-threshold compressive force (*f*_c_ = 20 pN), exerted via the FGN-bearing probe induced much higher level of JON/A binding to diabetic than control platelets (Fig. 6b). Importantly, platelets maintained their discoid morphology throughout the experiment indicating they remained in a low-activation state as observed in our in vivo thrombosis models and in vitro perfusion studies. The 3.2-fold higher adhesion frequency of JON/A to diabetic platelets translated into a 3.3-fold higher level of JON/A epitope exposure^52^. Interestingly, the below-threshold *f*_c_ = 10 pN did not induce JON/A binding enhancement in diabetic platelets (Fig. 6b). These data indicate that compressive force on diabetic platelets induces α_IIb_β_3_ activation. In contrast, stimulation by ADP resulted in much higher, but indistinguishable levels of JON/A adhesion frequencies to diabetic and non-diabetic platelets (Fig. 6b), suggesting that the enhanced integrin activation is specific to biomechanical stimulation rather than agonist stimulation.

To investigate the cumulative effect of compressive force over time, we performed a time-lapse adhesion frequency assay for a total of 100 repeated touches (at 2.5 s per cycle) on the BFP at *f*_c_ = 20 pN. The adhesion frequency was estimated for every 10 consecutive touches and plotted vs. elapsed time (=2.5 s × cycle number). The FGN adhesion frequency to non-diabetic platelets fluctuated around the baseline, probably due to a small number of stochastic events. In sharp contrast, the initially slightly higher FGN adhesion frequency to diabetic platelets increased by ~2-fold within the first 125 s and by another 50% in the following 125 s (Fig. 6c). As expected, replacing FGN with vWF-A1 on the BFP probe abolished the time-dependent adhesion increase (Fig. 6d), thus confirming that the phenomenon is integrin specific. Matching our mouse studies, a similar accumulation of FGN binding enhancement over time was also observed in diabetic, but not in non-diabetic human platelets (Fig. 6e). These observations suggest that the compression force effect on integrin α_IIb_β_3_ activation is cumulative in diabetic platelets.

The lack of compressive force effect on vWF–GPIb mediated adhesion on platelets from both diabetics and controls suggest that the differential FGN–integrin α_IIb_β_3_ on-rate enhancement by compressive force in diabetic platelets is due to a biological rather than biophysical mechanism (e.g., a compression-induced enlargement of the contact area between the platelet and the probe bead). This assertion was also supported by the observation that the frequency of fibrinogen adhesion to purified integrin α_IIb_β_3_ coated on a glass bead was not enhanced with increasing contact force (Supplementary Fig. 7). Moreover, the cumulative effect of compressive forces over time suggested that platelet activation may contribute to this process. Simultaneous calcium imaging in FGN perfusion experiments revealed that diabetic platelets displayed increased calcium signaling during platelet adhesion under shear (Fig. 6f), consistent with previous studies^9^. To test the hypothesis that the ligand binding on-rate enhancement of integrin α_IIb_β_3_ by compressive force involves platelet signaling, we performed the BFP compression assay in the presence of 1 mM Mg^2+^/EGTA to chelate extracellular calcium. EGTA did not significantly affect FGN adhesion frequency to non-diabetic platelets but reduced the adhesion frequency of diabetic platelets to the level seen in non-diabetic controls (Fig. 6g). These data suggest that the compressive force-induced differential fibrinogen adhesion to diabetic platelets requires extracellular calcium influx to support signaling.

### Compression force sensing activates integrin α_IIb_β_3_

**Fig. 7:**
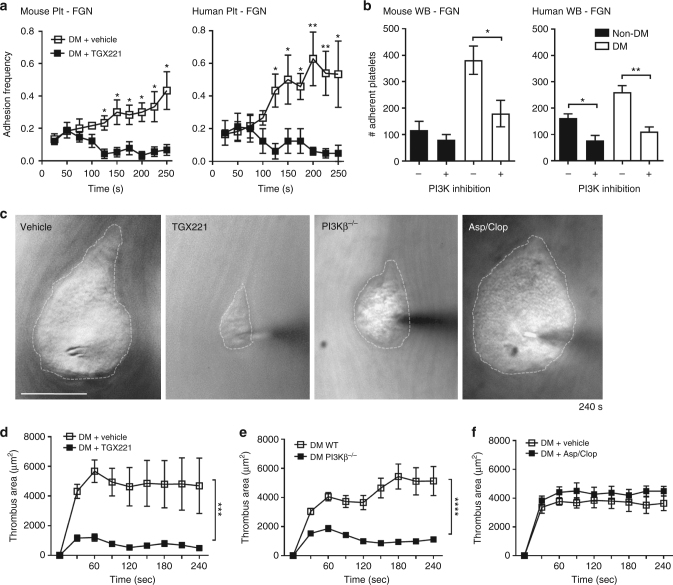
PI3Kβ regulates integrin α_IIb_β_3_ biomechanical signaling and adhesive function in diabetic platelets. **a** Washed platelets were isolated from non-DM and DM mice or humans, and treated with either DMSO (vehicle) or the PI3Kβ inhibitor TGX221 (TGX221, 0.5 µM), then subjected to 100 BFP cycles using FGN-coated beads, as described in Fig. 6c–e. Adhesion frequency was analyzed for every 10 consecutive cycles and plotted over time (2.5 s/cycle). **b** Hirudinated whole blood from non-DM and DM mice or humans were treated with DMSO (vehicle) or the pan-isoform PI3K inhibitor LY294002 (100 μM) for 10 min, perfused over FGN matrices at 600 or 300 s^−1^ for mouse or human blood respectively, and the number of adherent platelets analyzed after 4 min perfusion. **c**–**f** Thrombi were induced using the needle in situ model in diabetic mice treated with DMSO (vehicle), or PI3Kβ inhibitor TGX221 (2.5 mg kg^−1^), or aspirin/clopidogrel, and in diabetic PI3Kβ^−/−^ mice. **c** Representative DIC images depict thrombi (broken outline) formed around the needle tip 240 s post needle insertion. Note that the enhanced thrombotic response observed in diabetic subjects was abrogated by PI3Kβ deficiency or inhibition, but not by aspirin/clopidogrel. **d**–**f** Thrombus surface area was quantified over the entire 4 min period for the indicated diabetic mouse types. Results are expressed as the mean ± s.e.m. of *n* = 3 mice, with 6 (**d**) or 8 (**e**, **f**) thrombi/mouse. Results were assessed by an unpaired, two-tailed Student’s *t*-test, where *p < 0.5; **p < 0.01; ***p < 0.001; ****p < 0.0001; Scale bars = 50 μm

We have previously demonstrated an important role for PI 3-kinase (PI3K), especially the type 1 PI3Kβ isoform, in regulating integrin α_IIb_β_3_ signaling and adhesive function under shear^53^. PI3K signaling has also been demonstrated to be dysregulated in diabetic platelets^30^. We therefore examined the effect of the PI3Kβ isoform selective inhibitor TGX221 in the BFP time-lapse adhesion frequency assay at a 20-pN compressive force. TGX221 suppressed the increment in the fibrinogen adhesion frequency observed in diabetic platelets after 50 s (Fig. 7a). Similar effects of PI3K inhibition in abrogating the enhanced adhesion of diabetic platelets to FGN under shear was observed with diabetic mouse platelets (Fig. 7b). Importantly, the effects of PI3K inhibition were confirmed in human diabetic platelets in both BFP and perfusion assays (Fig. 7a, b). These findings suggest that PI3K inhibition abolishes integrin affinity maturation by disrupting cumulative compression force sensing, and therefore inhibits integrin α_IIb_β_3_-dependent enhanced diabetic platelet adhesion under shear.

To examine the impact of PI3Kβ on the exaggerated biomechanical thrombosis observed in diabetic mice in vivo, intravital needle in situ experiments were performed in mice treated with the PI3Kβ inhibitor TGX221. Notably, TGX221 treatment dramatically attenuated the rate and extent during the early phase of the discoid platelet aggregation (<60 s) in diabetic mice (Fig. 7c, d), with the aggregate surface area reduced by 80% at the peak growth time (60 s) (Fig. 7d). Furthermore, TGX221 treatment also reduced the stability of discoid platelet aggregates in diabetic mice (Fig. 7c, d and Supplementary Movie 2). Similar findings were also seen with PI3Kβ knockout (PI3Kβ^−/−^) mice rendered diabetic by streptozotocin injection (Fig. 7c, e and Supplementary Movie 2). In contrast, diabetic mice treated with the conventional anti-platelet agents, aspirin and clopidogrel, demonstrated no detectable changes in the formation and stability of discoid platelet aggregates in this model (Fig. 7c, f and Supplementary Movie 2). Together, these data confirm a critical role for PI3K in the dysregulation of the integrin α_IIb_β_3_ response to compressive force and the biomechanical adhesive function of discoid platelets.

## Discussion

How integrin α_IIb_β_3_ senses and responds to mechanical stimulation on platelets remains unclear, but is likely to be a fundamental issue in platelet biology given the central role played by this receptor in mediating platelet-endothelial and platelet–platelet interactions under the influence of hemodynamic forces. The studies presented here demonstrate the existence of a compression force sensing mechanism utilized by platelets to regulate the adhesive function of integrin α_IIb_β_3_. This mechanosensing mechanism is operative in discoid platelets, triggered by a threshold level of applied compressive force, and requires intracellular signaling events linked to the activation of integrin α_IIb_β_3_. Functionally, it is associated with the enhanced adhesion of discoid platelets to immobilized fibrinogen and vWF, and an exaggerated discoid platelet aggregation response in vivo. In contrast, agonist-induced increases in integrin α_IIb_β_3_ affinity were no different between diabetic and non-diabetic mouse platelets. This surprising finding was mirrored in vivo, where microinjection of either ADP or thrombin was not associated with an exaggerated thrombotic response in diabetic mice. Significantly, dysregulated biomechanical α_IIb_β_3_ activation is insensitive to the platelet inhibitory effects of aspirin and P_2_Y_12_ receptor antagonists, but is highly sensitive to inhibitors of α_IIb_β_3_ outside-in signaling, such as PI 3-kinase. These findings define a distinct diabetic prothrombotic mechanism linked to dysregulated biomechanical integrin α_IIb_β_3_ activation that may partly explain resistance to oral antiplatelet therapies.

In contrast to tensile and shear forces, which are far more extensively studied in mechanobiology, the biological effects of compressive force and the mechanisms of its reception, transmission and transduction in cells is poorly defined. Our study has provided the first evidence demonstrating that compression force sensing by platelets alters the adhesive behavior of integrin receptors. Insight into this was first provided from our in vitro flow assays where a critical role for RBCs was identified to differentially enhance the adhesive phenotype of diabetic platelets. To date, most studies have suggested that the primary effect of RBCs is to increase platelet transport (margination) from the blood stream to the vessel wall, thereby enhancing the probability of platelets forming adhesive contacts^42,44,45^. Notably, most studies make a common assumption that adhesion receptors on the platelet surface do not undergo marked changes during the RBC-dependent platelet margination, which may not be the case. It is likely that RBCs increase platelet compression against ligand surfaces to enhance the formation of nascent adhesive contacts. However, the molecular insights underlying the RBC “compressive effects” have been difficult to study, largely due to the technical challenge of applying single-molecule nanotools to live cells as small and reactive as platelets. The recent advancements in the BFP assay have enabled investigation of the single-receptor mechanosensing behavior on the surface of live platelets^54–56^. Using the BFP, we have demonstrated that a threshold force is required to enhance α_IIb_β_3_ engagement of immobilized fibrinogen and that the efficiency of adhesion was influenced by both the magnitude and repetitive nature of the applied compressive force.

Our use of repetitive compression cycles in the BFP assays is a reductionist approach to model platelet translocation dynamics, wherein individual platelets continuously form and break bonds. It is likely that translocating platelets experience continuous compression forces due to frequent collisions with RBCs in the bulk flow^44,45^. However, direct measurement of compression force on a platelet in free flow is technically challenging. For a platelet tethered to a surface by an adhesive contact at the rear end, we have performed a calculation as previously published^57,58^. For the venous to arterial shear rates studied in this report, *γ* = 600, 1800, and 5000 s^−1^, we estimated the compression forces *f*_c_ = 11–24, 35–73, and 98–202 pN, respectively, relevant to those tested in the BFP experiments (see Supplementary Note). However, the precise relationship between the compression forces applied in our BFP assays and those experienced by platelets in vivo will be an important question for future investigation.

How compressive forces exerted on the platelet membrane leads to integrin α_IIb_β_3_ activation will also be an important question for future studies but we postulate multiple potential mechanisms. The first possibility is in accordance with the force-through-lipid model which has been established for mechanosensitive ion channels i.e., MscL^59^. In this scenario, compressive force normal to the membrane is converted into tension in the membrane, which may trigger the opening of Ca^2+^ ion channels and induce integrin activation. This is consistent with our observation that chelating extracellular calcium abolished α_IIb_β_3_–FGN adhesion enhancement on diabetic platelets. The second possibility may be related to the force-through-filament principle as the compressive force is sensed by the cytoskeleton rather than plasma membrane. In response to the external compressive force, the platelet cytoskeleton might undergo local remodeling, leading to integrin activation. Indeed, compressive force has been shown to alter the growth of branched actin-filaments at the leading edge of crawling cells^60^. Recent experimental and modeling studies have shown that integrin inside-out activation requires the cytoskeletal force to be transmitted through talin to the cytoplasmic tail of the β subunit^61–63^. An additional possibility may be related to the glycocalyx, a layer of glycoprotein–polysaccharide complex on the cell surface, since a recent study has demonstrated that local compression of the glycocalyx near integrin adhesive contacts promotes integrin clustering and focal adhesion maturation^64^.

The demonstration that chronic hyperglycemia in the mouse primarily resulted in a normal platelet response to agonist stimulation was surprising, since many biochemical changes associated with human diabetes can be recapitulated in rodent diabetes models^31^. Consistent with this, we have found dysregulated calcium flux and PI 3-kinase signaling in our STZ-diabetes mouse model, verifying previous findings^9,30^. The STZ-diabetes mouse model also exhibits other important diabetes-related pathogenic mechanisms, including increased oxidative stress and the generation of advanced glycation end products^17^. A previous study using the STZ model of diabetes has reported an enhanced platelet aggregatory response to low concentrations of ADP and PAR4-AP, in conjunction with augmented signaling processes downstream of their respective receptors^30^. The discrepancy between our findings and those by Stolla et al.^30^ are not immediately apparent, although presumably are related to methodological differences. The studies presented here were performed using mice with confirmed hyperglycemia of 7–10 weeks in duration, whereas the hyperglycaemic duration in studies by Stolla et al.^30^ was not specified. It is entirely possible that there may be mouse strain, as well as species-specific differences in platelet responsiveness to chronic hyperglycemia. It is also possible that exaggerated platelet responses in human platelets (particularly in type 2 diabetes) may occur at least in part as a result of additional cardiovascular risk factors, such as dyslipidemia and hypertension. An interesting possibility that warrants further investigation is whether the duration of hyperglycemia has a major influence on the sensitivity of platelets to mechanical or chemical stimuli. In this context, most studies in humans have employed subjects with chronic hyperglycemia over many months and years, so it will be important to investigate time-dependent changes in platelet reactivity soon after diabetes diagnosis, particularly in patients with type 1 diabetes. Nonetheless, our demonstration that dysregulated biomechanical integrin α_IIb_β_3_ activation occurs in platelets from both STZ-treated mice and humans with type 1 diabetes, independent of other cardiovascular risk factors, suggests that this process is primarily induced by biochemical changes associated with chronic hyperglycemia.

How compressive force sensitivity relates to the many aspects of platelet hyperactivity and dysfunction reported in diabetes remains an important issue for future investigation. Some of the platelet abnormalities in diabetes involve receptors or signaling pathways linked to platelet activation by soluble agonists, most notably TxA_2_ and ADP^35^ and collagen exposure^34^. This may partly explain the requirement for higher doses of aspirin or clopidogrel to reduce thrombosis risk in diabetes^21^. Our demonstration of dysregulated biomechanical integrin α_IIb_β_3_ activation in diabetic platelets may have direct clinical relevance, since it may partly explain the effectiveness of integrin α_IIb_β_3_ antagonists in diabetic individuals. Our findings from multiple distinct experimental approaches, including the calcium flux assay under shear, BFP time-lapse adhesion assay and FGN-to-JON/A ‘switch’ assay are consistent with the possibility that integrin downstream signaling is dysregulated in diabetic platelets, leading to an exaggerated discoid platelet adhesion response. In this regard, the lipid kinase PI3K, especially its β isoform, which is an important mediator of α_IIb_β_3_ outside-in signaling has previously been demonstrated to play a key role in integrin α_IIb_β_3_-dependent platelet adhesion^53^. Indeed, inhibition of PI3K markedly inhibits α_IIb_β_3_ adhesion maturation and α_IIb_β_3_-mediated platelet adhesion under shear. In contrast, PI3K does not appear to have a significant role in initiating α_IIb_β_3_ activation following soluble agonist stimulation, but rather, sustains α_IIb_β_3_ activation through regulation of Rap1b and AKT^53,65^. Furthermore, PI3K has been demonstrated in other cell types to play an important role in regulating the activation of integrin α_v_β_3_ in response to biomechanical force^66^. Given the effectiveness of PI3K inhibition in preventing biomechanical platelet activation in diabetes, therapeutic targeting of integrin α_IIb_β_3_ signaling pathways may represent an innovative approach to reduce the prothrombotic effects of diabetes.

## Methods

### Mouse strains

All procedures involving the use of mice for both in vitro and in vivo studies were approved by the Alfred Medical Research and Education Precinct (AMREP) Animal Ethics Committee (Melbourne, Australia) (project # E/1068/2011/M and E/0856/2009/M), and the University of Sydney Animal Ethics Committee (Project # 2014/620). All C57BL/6J male mice were sourced from AMREP animal services or Australian BioResources (ABR, Moss Vale, NSW, Australia). The PI3Kβ-deficient (PI3Kβ^−/−^) mice were obtained from the Jackson Laboratory (Bar Harbor, ME, USA).

### Generation of mice with type 1 diabetes mellitus

Diabetes was induced in 6-week-old male C57BL/6J mice via daily intraperitoneal injection of streptozotocin (STZ, 55 mg kg^−1^) for 5 consecutive days^67^. Blood glucose levels were examined 1 week post STZ injection, and monitored during their maintenance and prior to experiments. Mice which had blood glucose levels >15 mM were considered diabetic. Diabetic mice 7–10 weeks post STZ injection were used in this study, unless indicated otherwise. Diabetes was also induced in 6-week-old male PI3Kβ^−/−^ mice and hyperglycemia was confirmed using the same procedures.

### Patients with diabetes

Blood was obtained from patients with type 1 diabetes from The Diabetic Clinic, Alfred Hospital (Melbourne, Australia) with the approval of the Alfred Health Ethics Committee (project # 388/11 and 409/14). Age and gender matched non-diabetic healthy donors were also recruited as controls. All participants were confirmed devoid of any anti-platelet medication for the preceding 2 weeks, and were moreover free from any history of bleeding disorder, anemia, renal impairment, and acute coronary syndrome or coronary intervention (<30 days). Patients with type 2 diabetes were excluded from this study. The total number of participants enrolled in these studies included 24 diabetic patients (12 males, 12 females, age range 20–64 years old) and 24 non-diabetic healthy donors (12 males, 12 females, age range 33–57 years old).

Juvenile type 1 diabetic patients were recruited under the approval of the Emory University Ethics Committee (Protocol number H14444 at Georgia Tech and IRB00077577 at Emory University) and the studies on these patients were conformed to the principles outlined in the Declaration of Helsinki. Blood was obtained from these patients in the Emory Children’s Center on the Emory University campus. All participants were aged between 7–19 years old, and did not have a history of bleeding disorder, anemia, renal impairment, and acute coronary syndrome or coronary intervention (<30 days). All participants and their accompanying guardians were provided with a verbal explanation of the study. A verbal agreement and a signed consent form were respectively collected from the participants and accompanying guardians, stating that they agree for blood donation, and for the publication of the data collected using their blood. The participants in this cohort included 3 non-DM individuals (2 males, 1 female, age range 9–12 years old) and 5 DM patients (3 males, 2 females, age range 7–18 years old). In all cases, no fasting was requested prior to blood donation.

### In vitro platelet adhesion under flow conditions

For perfusion studies using mouse whole blood, nitric acid-washed glass microslides (0.1 × 1.0 mm: height × width)(Vitrocom; Mountain Lakes, NJ, USA) were coated with fibrinogen (FGN; 100 μg mL^−1^) for 2 h at room temperature, or overnight at 4 °C, then blocked with 20 mg mL^−1^ BSA for 30 min. Hirudinated mouse whole blood was perfused through the FGN coated microslides at 600 s^−1^ for 5 min. Platelet adhesion was monitored using an inverted Leica DMIRB microscope and differential interference contrast (DIC) microscopy (×63, NA 1.2 water objective). Platelet adhesion was recorded using Micro-manager v1.4 for off-line analysis. For perfusion studies with human whole blood, mouse washed platelets or reconstituted blood, platelets were perfused through FGN-coated microslides (100 μg mL^−1^; 0.2 × 2.0 mm) at 200 or 300 s^−1^ for 5 min. For mouse blood perfusion studies using murine vWF matrix or spread platelet monolayers, polydimethylsiloxane (PDMS) microfluidic channels (0.3 × 0.1 mm (height)) were used^68^. To capture vWF, the bottom of PDMS device was coated with an anti-vWF antibody AB7356 (20 μg mL^−1^; Merck Millipore) for 1 h at room temperature, then incubated with mouse plasma for 2 h at room temperature to capture vWF^69^. After the BSA blockade for 0.5 h, the mouse blood was perfused through at preset shear rates (i.e., 600, 1800 and 5000 s^−1^). To mitigate the pre-activation effect of microfluidics on platelets, the hirudinated blood was pre-treated with amplification loop blockers (ALB): ADP receptor blockers MRS2179 (100 μM), 2-MeSAMP (10 μM), cyclooxygenase inhibitor indomethacin (10 μM).

Platelet adhesion was analysed off-line using ImageJ 1.50a (Fiji). Platelets were considered adherent if they remained attached to the matrix for at least 2 s. Platelets which moved <1 cell diameter in 10 s following tethering to the matrix were classified as stationary cells, and platelets that rolled (continuously or stop/start) over a 10-s period were considered rolling cells.

### Biomembrane force probe compression assay

The BFP utilizes two micropipettes, one aspirating a RBC with a glass bead attached on the apex via biotin-streptavidin interaction to serve as a force transducer^52,54,55^. Its spring constant of *k*_BFP_ = 0.15–0.3 pN nm^−1^ is determined by the aspiration pressure and the radii of the micropipette and the RBC-bead contact area^47,70^. The glass bead, also called “probe” bead, was coated with FGN, vWF-A1 or JON/A via maleimide-PEG3500-NHS (MW ~3500; JenKem, TX). The other micropipette aspirates a platelet and dives the platelet to contact the probe under a defined compressive force (*f*_c_ = 5–40 pN) for a certain contact time (*t*_c_) (0.2 s by default, 0.1–5 s for affinity measurement) to allow bond formation. The platelet is then retracted at a constant speed (3.3 μm s^−1^) for bond detection. During the retraction phase, a bond formation is signified by a tensile force. Both compressive and tensile forces are measured by quantifying the RBC deformation, which is detected by the axial movement of the probe bead tracked by a high-speed camera. Each aspirated platelet is subjected to repetitive cycles (50 by default, 100 for time-lapse measurement) of touch with the probe bead, and the fraction of bond formation over the cycles was determined as adhesion frequency *P*_a_.

To assess the kinetics of the bond formation, the aspirated platelet was allowed to contact the ligand coated bead for various durations, since the adhesion frequency *P*_a_ depends on the contact time (*t*_c_). The 2D zero-force cellular on-rate (*m*_r_*m*_l_*A*_c_*k*_on_) and off-rate (*k*_off_) can be derived by fitting the *P*_a_ vs. *t*_c_ curve with the model *P*_a_ = 1 − exp{−*m*_r_*m*_1_*A*_c_*k*_on_[1 − exp(−*k*_off_*t*_c_)]/*k*_off_}, where *A*_c_ is the contact area, assumed to be constant for a given compression force. The site densities of integrin α_IIb_β_3_ (*m*_r_) on mouse platelets and fibrinogen (*m*_l_) on beads were measured as *m*_r_ = 681 – 2098 µm^−2^ for non-DMs and 969–2262 µm^−2^ for DMs and *m*_l_ = 90 µm^−2^, using a flow cytometry method with the Quantum™ FITC-5 MESF (555p, Premix) calibration beads from Bangs Laboratories (Fishers, IN, USA)^47^. The signals were detected by the FITC labeled anti-α_IIb_β_3_ mAb (Leo.F2) from Emfret Analytics (Wurzburg, Germany) and the anti-fibrinogen γ chain mAb (LS-C418056) from LifeSpan BioSciences (Seattle, WA, USA), respectively. Then we divided the measured cellular on-rate parameters by these site density numbers to obtain the effective 2D on-rates (*A*_c_*k*_on_). The effective 2D affinity can be calculated from *A*_c_*K*_a_ = *A*_c_*k*_on_/*k*_off_.

Of note, for adhesion frequency analysis to extract 2D kinetics, we made no claim on whether the measurements were done under the conditions of single molecular interactions. Such claim is not needed because at higher adhesion frequencies the measurements were not made under single bond conditions and because such conditions are not needed for our conclusions on affinity change and on-rate changes^49^. Moreover, the 2D kinetic rates we measured are on the condition that the integrins are NOT subject to tensile force. Whereas the platelets were subject to various compression forces. The changing integrin affinity is a biological response of the platelet that involves signaling.

### Models of thrombosis in vivo

C57BL/6J control or diabetic male mice with a body weight of 18–25 g were subjected to various in vivo thrombosis models. All mice were anesthetized with sodium pentobarbitone (60 mg kg^−1^) or ketamine/xylazine (150/15 mg kg^−1^). The anesthetized mice were provided with oxygen (0.5 L min^−1^) via a mask during the entire experimental procedure.

### Needle in situ model and intravital microscopy

Thrombi were induced in the mesenteric venules or arterioles of diabetic and non-diabetic mice using the needle in situ model^33^. Microinjector needles were fashioned using glass capillaries (GD-1, 50 mm; Narishige) with a micropipette puller (PC-10; Narishige, Tokyo, Japan) such that the needle tip had a diameter of 2–3 µm. The needle was inserted into the lumen of mesenteric vessels (100–200 µm in diameter), with the needle tip positioned ~20 µm from the base of the vessel using an Injectman micromanipulator (Eppendorf, Hamburg, Germany). Thrombus formation at the needle tip was monitored for 4 min under real-time DIC microscopy using an Olympus IX81 inverted microscope with a ×40 objective, and recorded via EM-CCD camera (QuantEM 512SC; Photometrics, Tucson, USA) for off-line analysis using Visiview software 2.1.4 (Visitron System GmbH, Germany). Thrombus surface area at the indicated times was determined using ImageJ software. To examine platelet P-selectin expression during thrombus formation, mice were preadministered DyLight 649-anti-GPIbβ Ab (100 µg kg^−1^) and Alexa 546-anti P-selectin Ab (50 µg kg^−1^). P-selectin expression and thrombus formation were monitored using Nikon A1R confocal intravital microscopy with a ×40 objective. Fluorescent images were captured using NIS software, Version 4 (Nikon, Japan). Where indicated, mice were pre-treated with the anti-platelet agents aspirin (oral, 200 mg kg^−1^) and clopidogrel (oral, 3 mg kg^−1^) or vehicle (Gum Arabic) 24 h and 2 h prior to experimentation. Mice were also treated with the PI3Kβ inhibitor TGX221 (2.5 mg kg^−1^) or vehicle (DMSO), 10 min prior to needle in situ thrombosis. To inhibit integrin α_IIb_β_3_ ligand binding, mice were infused intravenously with integrilin (4 mg kg^−1^) via a carotid catheter every 15 min during the entire experimental period.

### Needle injury thrombosis model

Thrombi were induced at sites of mesenteric venule injury via needle puncture^33,37^. Thrombi were created by repetitive puncture (×10) of the mesenteric venules using a needle that had a 2 µm tip and was controlled by an Injectman micromanipulator. Thrombus formation at sites of vessel injury was monitored using intravital DIC microscopy, and thrombus surface area determined using Image J. Where indicated, the thrombi were subjected to local microinjection of ADP (10 mM) or thrombin (100 U mL^−1^) by positioning the microinjector needle, ~40 µm upstream of the thrombi in the direction of blood flow. The agonist injection was carried out under a prespecified pressure (1000 kPa) and duration (1.5 s) for 10 times. Of note, the needle does not breach or denude the endothelium. The thrombin was only locally injected, 20–40 µm upstream of the thrombus, not to the entire venule, and the quantity injected was very small (0.14 nL). Local thrombin injection also served as a positive control for induction of P-selectin expression in the needle in situ thrombosis model presented in main Fig. 1b, right panel.

### Mechanical crush injury of mesenteric venules

A microinjecter needle tip was blunted (30–50 μm) and positioned above the exposed mesenteric venule. The mesenteric venule was then crushed twice by the blunted needle using the Injectman micromanipulator. Platelet adhesion to the crushed endothelial area was monitored at real time using DIC microscopy. Where indicated, mice were systemically injected with anti-PECAM Ab (FITC, 0.2 mg kg^−1^), Alexa-488 Annexin V (0.1 mg kg^−1^), GPIbβ Ab (DyLight 649, 100 μg kg^−1^), or P-selectin Ab (Alexa 546, 50 μg kg^−1^) to monitor endothelial cells, endothelial cell death, platelet adhesion, and activation, respectively. In order to examine ICAM-1-depedent fibrinogen deposition to the crushed endothelium, mice were pre-injected with Alexa-488-fibrinogen (17 mg kg^−1^) in the absence or presence of the ICAM-1 blocking Ab (YN1, 200 μg per mouse). To assess α_IIb_β_3_-dependent platelet adhesion, mice were injected with integrilin (4 mg kg^−1^) every 15 min Following crush injury, DIC, fluorescence and confocal intravital microscopies were performed as described under the ‘needle in situ’ thrombosis model. For platelet adoptive transfer studies, platelets were isolated from non-diabetic and diabetic donor mice, labeled with Alexa 488-anti-GPIbβ Ab, and resuspended in Tyrode’s buffer at a concentration of 1 × 10^9^ mL^−1^. The recipient non-diabetic mice were sytemically administered DyLight 649-anti-GPIbβ Ab (100 μg kg^−1^) to label endogenous platelets, and then infused with donor platelets via inferior vena cava to achieve a donor/recipient ratio of 1/10. The mesenteric venules of the recipient mice were then subjected to mechanical crush injury. The adhesion of infused non-DM, and DM donor platelets and recipient endogenous platelets was monitored using confocal intravital microscopy, and images taken pre- and 10 min post-injury. The fluorescence intensity of the circulating endogenous and infused donor platelets pre-crush, and those adherent to the endothelium post-crush were quantified using ImageJ, and expressed as the ratio of donor over recipient platelets.

### Data availability

The data that support the findings of this study are available from the corresponding author upon reasonable request.

## Electronic supplementary material

Supplementary Information

Description of Additional Supplementary Files

Supplementary Movie 1

Supplementary Movie 2
