# Supplementary material for: Compression force sensing regulates integrin αIIbβ3 adhesive function on diabetic platelets
Source: Nat Commun. 2018 Mar 14;9:1087. doi: 10.1038/s41467-018-03430-6 (PMC5852038; doi:10.1038/s41467-018-03430-6)
Supplement: Supplementary file 1 — Supplementary Information [file 41467_2018_3430_MOESM1_ESM.pdf]

SUPPLEMENTARY INFORMATION

Compression Force Sensing Regulates Integrin $\alpha_{IIb}\beta_3$ Adhesive Function on Diabetic Platelets

Authors: Lining Ju et al.

SUPPLEMENTARY METHODS

Mouse Strains

All procedures involving the use of mice for both *in vitro* and *in vivo* studies were approved by the Alfred Medical Research and Education Precinct (AMREP) Animal Ethics Committee (Melbourne, Australia) (project # E/1068/2011/M and E/0856/2009/M), and the University of Sydney Animal Ethics Committee (Project # 2014/620). All C57BL/6J male mice were sourced from AMREP animal services or Australian BioResources (ABR, Moss Vale, NSW, Australia). The PI3Kβ-deficient (PI3Kβ$^{-/-}$) mice were obtained from the Jackson Laboratory (Bar Harbor, ME, USA).

Human blood collection and platelet isolation

All procedures involving the collection of blood from healthy non-diabetic donors were approved by the Monash University Standing Committee on Ethics into Research Involving Humans (SCERH, Project CF07/0141-200700005), the University of Sydney Human Research Ethics Committee (HREC, Project 2014/244) and the Institutional Review Board of the Georgia Institute of Technology (project number H12354). Blood was drawn via venesection and anticoagulated as indicated. All human donor blood samples were obtained with informed consent.

For whole blood perfusion studies, blood was anticoagulated with hirudin (800 U mL^{-1}). For platelet isolation, blood was anticoagulated with acid-citrate-dextrose (ACD; 85 mM sodium citrate, 72.9 mM citric acid, 110 mM D-glucose, 70 mM theophylline) at a volume ratio 6:1 (blood:ACD). Platelets were then isolated and resuspended in Tyrode's buffer (pH 7.2-7.4; 12 mM NaHCO3, 10 mM Hepes, 137 mM NaCl, 2.7 mM KCL, 5.5 mM D-glucose) containing 1mM Ca^{2+}, 0.5% (v/v) BSA and apyrase 0.02 U mL^{-1} as previously described[1]. Platelet count was controlled at 3×10^8 mL^{-1} by default and read with the Sysmex KX-21N haematology analyser (Kobe, Japan). Isolated platelets were rested for 30 min at 37 ºC prior to use for experiments and utilized within 4 hrs of isolation.

Mouse blood collection and platelet isolation

Mice were anaesthetised with either sodium pentobarbitone (60 mg kg^{-1}) or ketamine/xylazine (150/15 mg kg^{-1}). Mouse blood was then collected via inferior vena cava, and platelets isolated and maintained as previously described[2].

Red blood cell preparation

Red blood cell (RBC) isolation and platelet reconstitution with RBCs were carried out as described previously[1]. Briefly, ACD anticoagulated whole blood was centrifuged at 300 g for 15 min to remove platelet rich plasma, and the RBC layer was centrifuged at 1,500 g for 10 min to remove plasma. The pelleted RBCs were washed twice with Tyrode's buffer. For perfusion experiments using platelets reconstituted with RBCs, the RBC pellet was treated with apyrase (0.02 U mL^{-1}) for 1 min then mixed with an equal volume of isolated platelets (50%)(v/v) with a final platelet count of 3×10^8 mL^{-1}. For BFP assays, human RBCs were biotinylated via biotin-PEG3500-SGA polymer (JenKem Technology; Plano, TX, USA) using the published protocol[3,4].

Analysis of cytosolic calcium flux in platelets

Intraplatelet calcium levels were monitored as previously described[5,6]. Briefly, washed platelets were loaded with two calcium indicator dyes, Oregon Green (OG)-488 BAPTA-1, AM (1 μM, emission wavelength 500-570 nm) and Fura Red (FR)-AM (1.25 μM, emission wavelength 600-710 nm) for 30 min at 37ºC. Dye-loaded platelets were resuspended in Tyrode's buffer containing 1 mM Ca^{2+}, reconstituted with RBCs and perfused over fibrinogen matrices at 600 s^{-1}. The ratio changes in OG and FR fluorescence during platelet adhesion to fibrinogen were monitored via confocal microscopy using Leica TCS NT software. The images were captured at a scanning rate of about half frame per sec over a period of 100 frames. Platelets demonstrating one calcium spike or more within 100 frames were considered as '*platelets with calcium flux'*.

Platelet adhesion under static conditions

Static adhesion assays were performed as previously described[1,6]. HMDS-treated glass coverslips were coated with fibrinogen (10 μg mL^{-1}) for 2 hr at room temperature or overnight at 4 ºC, and then blocked with BSA (20 mg mL^{-1}) for 30 min at room temperature. Platelets (1 x 10^7 mL^{-1}) were then allowed to adhere to fibrinoigen for 5 min at 37 ºC in a humidified chamber. Platelet

adhesion was monitored using DIC microscopy and the number of adherent platelets analysed at 5 min adhesion time.

$\alpha_{IIb}\beta_3$ expression and activation as well as P-selectin expression

The surface expression levels of $\alpha_{IIb}\beta_3$ on non-diabetic and diabetic platelets were assessed using a Calibur flow cytometer (Becton Dickinson; San Jose, CA, USA). Anticoagulated whole blood was diluted at 1/20 with Tyrode's buffer, and 50 µL of the diluted whole blood was incubated for 30 min with FITC conjugated Abs against $\alpha_{IIb}\beta_3$ (Leo.F2 for mouse and HIP8 for human), and then subjected to flow cytometry analysis. The fluorescent intensities (Geomean) were analysed using the FlowJo software (FlowJo LLC; Ashland, OR, USA).

To examine soluble agonist stimulated P-selectin expression and $\alpha_{IIb}\beta_3$ activation, diluted whole blood or isolated platelets were stimulated with PAR4 agonist peptide (PAR4-AP) (100 µM and 300 µM) or ADP (1, 3 or 10 µM) for 10 min in the presence of Abs against activated integrin $\alpha_{IIb}\beta_3$ (JON/A-PE for mouse) or P-selectin (RB40.34-FITC for mouse). The platelets were then fixed with 2% paraformaldehyde (PFA) and subjected to flow cytometry analysis. If not stated, the mouse Abs are from Emfret Analytics (Wurzburg, Germany) and human Abs are from BD Biosciences.

Platelet aggregation

Platelet aggregation was performed using non-diabetic and diabetic mouse platelet rich plasma. Platelet aggregation was induced by the indicated concentrations of ADP. The extent of platelet aggregation was monitored using an automated AggRAM analyser (Helena Laboratories; Tyne and Wear, UK).

Statistical analysis

Power calculations were used to establish sample size for in vivo animal experiments (significance level = 0.01; statistical power set at 80%). If statistical significance was reached with fewer animals, no additional animals were utilized. Statistical significance between 2 treatment groups was analyzed using an unpaired Student *t* test with two-tailed P values (Prism software v6.07; GraphPAD Software for Science, San Diego, CA). Data are usually presented as the mean ± s.e.m. of $n \geq 3$ independent experiments.

SUPPLEMENTARY NOTE

Estimation of the compression force of a tethered platelet under shear

For a platelet tethered to a surface by a single bond at the rear end, we performed a calculation as previously published[7,8]. The tether force (f_t) is balanced by the hydrodynamic drag force (f_s) and the compressive force (f_c) (Supplementary Fig. 8). Approximating the platelet by a sphere of radius r (=1μm), the hydrodynamic drag force has been estimated as $32 \times r^2 \times \gamma$, where γ is the shear rate of the bulk flow[7]. The compressive force is related to the drag force by $f_c = f_s \tan\alpha$ where α is the tether bond angle (=10-20 deg considering the platelet's discoid shape). For the three shear rates studied, γ = 600, 1,800 and 5,000 s^{-1}, at α = 10 deg, the f_c values were estimated to be 11, 35.3 and 98.2 pN; at α = 20 deg, f_c = 24.3, 72.9 and 202.6 pN. These estimates indicate that the range of compression forces tested in our BFP experiments are physiologically relevant.

SUPPLEMENTARY FIGURES

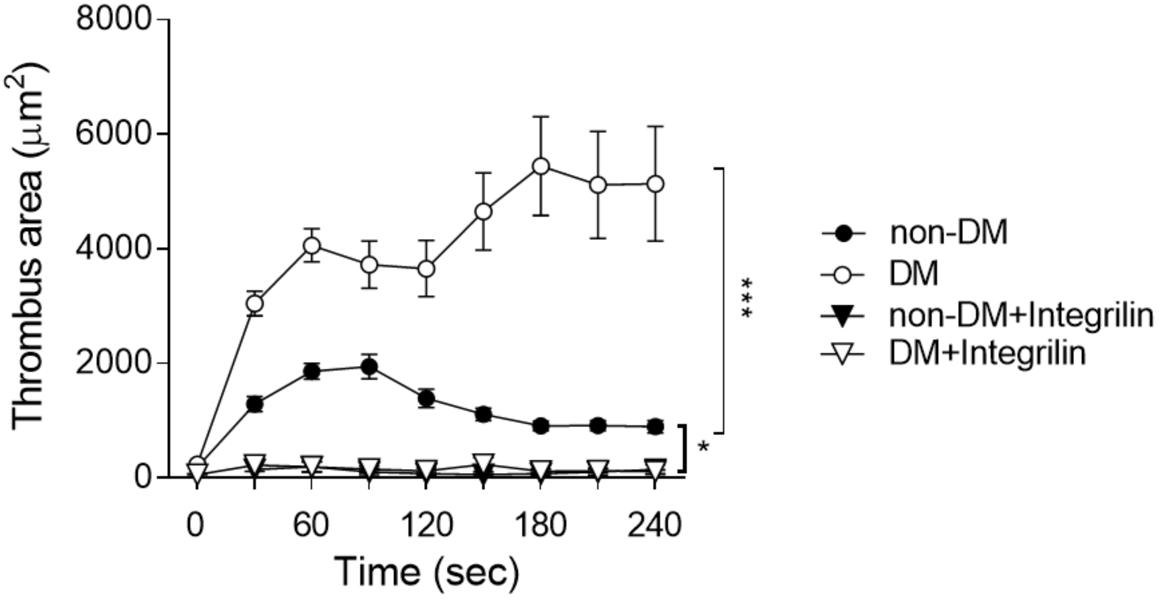

Supplementary Figure 1. Integrin $\alpha_{IIb}\beta_3$ blockade abolishes shear dependent enhancement of platelet aggregation *in vivo* in diabetic mice [corresponding to Fig. 1c,d]. Non-DM and DM mice were administered the integrin $\alpha_{IIb}\beta_3$ antagonist integrilin (4 mg kg^{-1}, i.v.), and mesenteric veins subjected 'needle *in situ*' thrombosis. ***Note* -** non-DM and DM untreated data sets (open and closed circles) are identical to data appearing in Fig. 1c. They are replicated here for comparison. The thrombus surface area of non-DM and DM mice was quantified at the indicated time points post-needle insertion. Results are presented as mean ± s.e.m. of n=3 mice (6-8 thrombi per mouse examined).

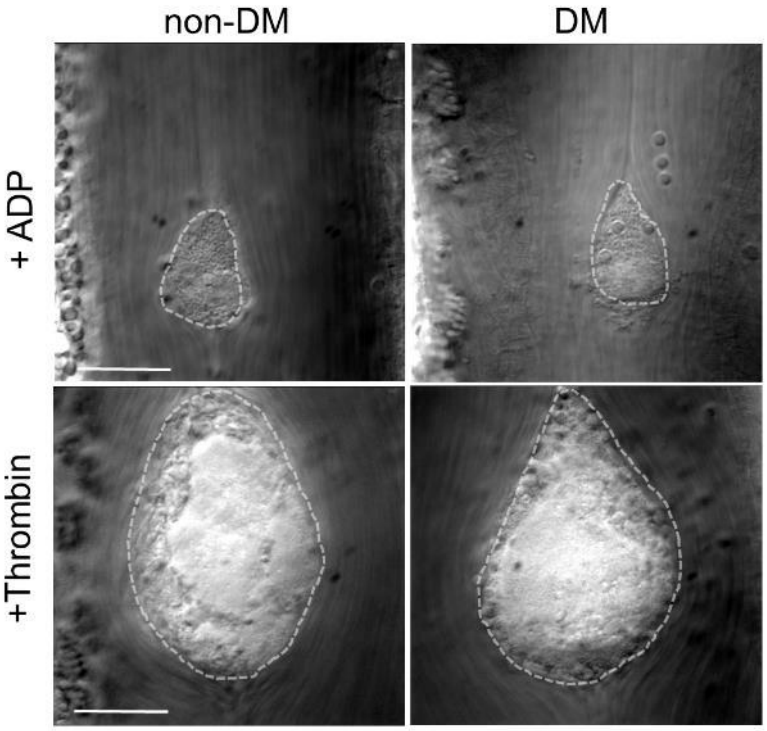

Supplementary Figure 2. Diabetic platelets do not display an enhanced thrombotic response to soluble agonists *in vivo* [corresponding to Fig. 1d-f]. Thrombi were induced in non-DM or DM mice by needle puncture of mesenteric veins, followed by immediate local microinjection of ADP (10 µM) or thrombin (100 U mL^{-1}). Thrombus formation was monitored by DIC microscopy. The representative DIC images depict the formed thrombi (marked by dotted lines). Images are from one representative of n ≥ 3 mice (3-4 injuries per mouse). Scale bars = 50 µm.

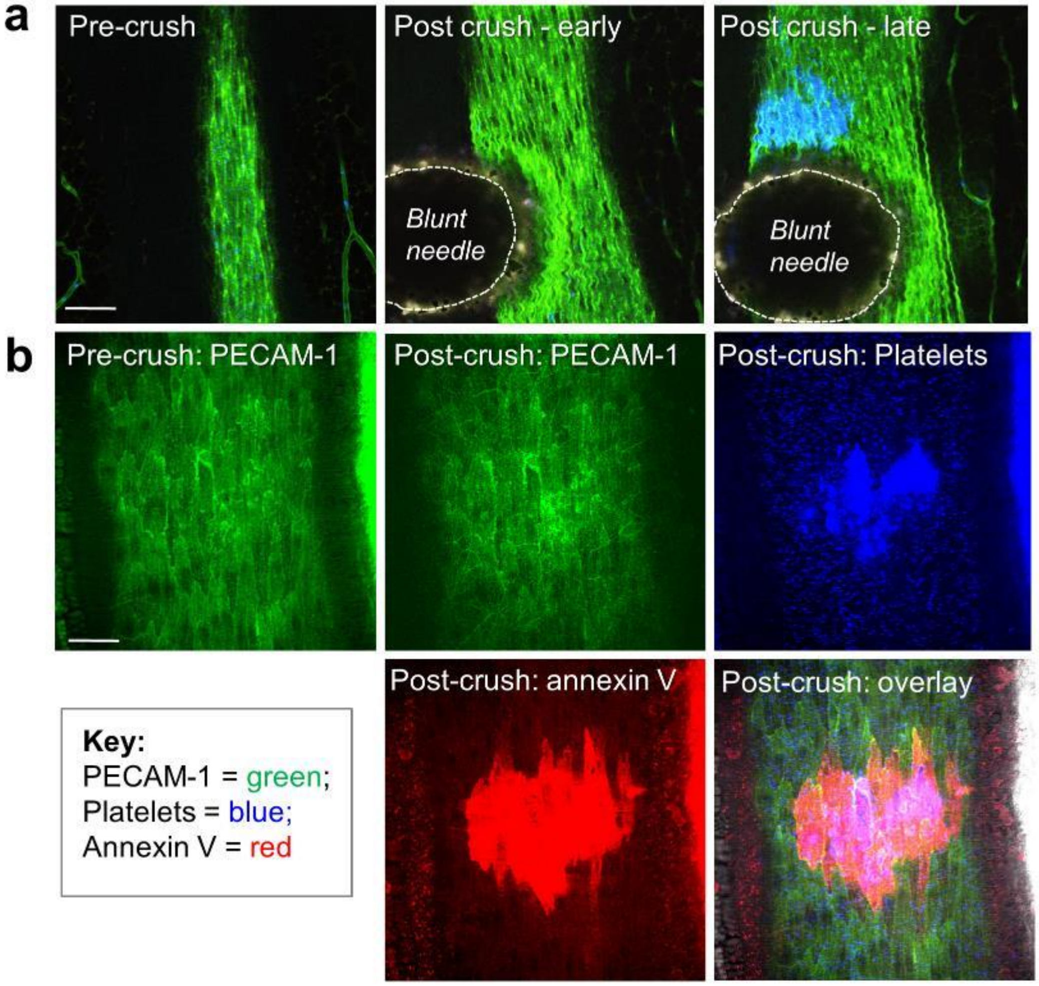

Supplementary Figure 3. Characterization of mesenteric vascular injury by needle crush [corresponding to Fig. 2]. Mouse mesenteric veins were subjected to 2 crushes by blunt needles as detailed in 'Methods'. Prior to crush injury, FITC-anti-PECAM-1 Ab, DyLight 649-anti-GPIbβ Ab and Alexa 488-annexin V were systemically administered to monitor endothelial cells, platelets and phosphatidylserine exposure, respectively. The injured endothelial areas were monitored using intravital confocal microscopy. (**a**) Representative confocal images showing the endothelium (green) pre (left), during (middle) and post (right) crush, with the blunt needle appearing as a dark ring (marked by dotted line). Note platelet adhesion (blue/cyan) post crush injury (right). (**b**) Representative single or overlay confocal fluorescence images showing the endothelial cells (green) pre- and post-crush injury, and platelet deposition (blue) to crushed endothelial cells and PS exposure (red) on endothelial cells. All images represent $n \geq 4$ mice (4-5 injuries per mouse). Scale bars = 50 μm.

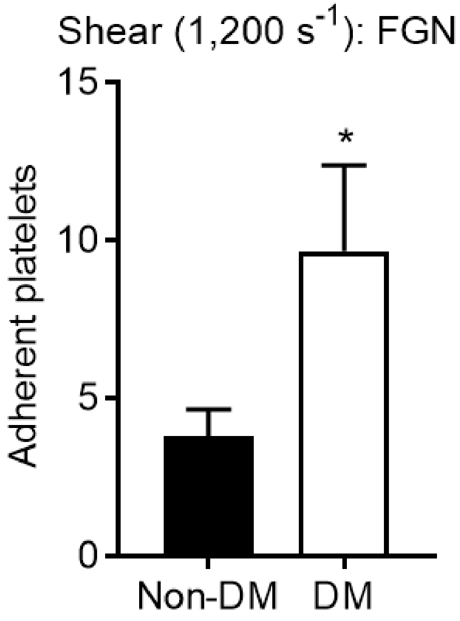

Supplementary Figure 4. Diabetes enhances integrin $\alpha_{IIb}\beta_3$ mediated platelet adhesion under high shear [corresponding to Fig. 3a]. Anticoagulated whole blood from non-DM or DM mice was perfused through fibrinogen (FGN) matrices at 1200 s^{-1} and the number of adherent platelets were determined at 2 min perfusion time. Results are presented as mean ± s.e.m. of n =3-5 mice with each perfusion performed in duplicates. * $p<0.05$.

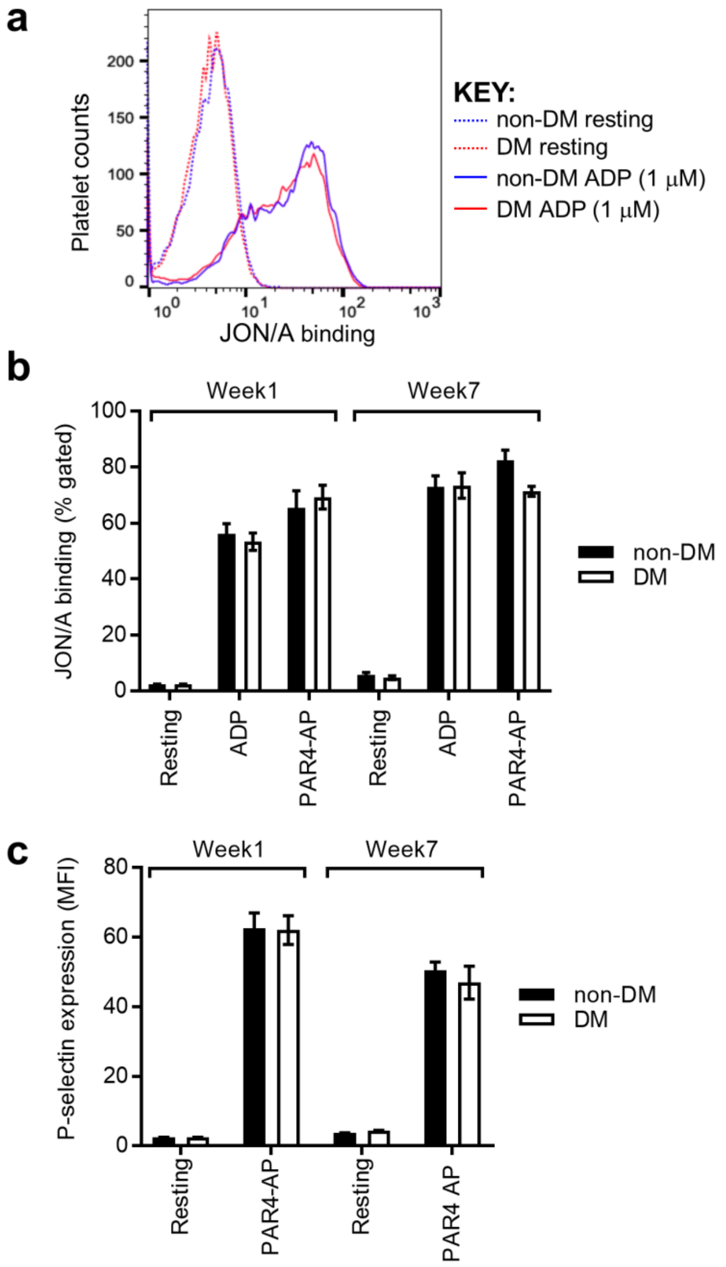

Supplementary Figure 5. Diabetic platelets do not display enhanced responses to soluble agonists [corresponding to Table 1]. Hirudin-anticoagulated whole blood from non-DM and DM mice at 1 and 7-week post STZ injection was kept unstimulated (Resting), or stimulated with indicated agonists for 10 min in the presence of PE-JON/A Ab (**a,b**) or PE-anti-P-selectin Ab (**c**), and then subjected to flow cytometry analysis. (**a**) Representative histograms showing threshold level ADP (1 µM) induced JON-A binding to non-DM and DM-platelets. (**b**) Platelet JON/A binding and (**c**) P-selectin expression in non-DM and DM platelets following ADP (10 µM) or PAR4-AP (300 µM) simulation. All results (mean ± s.e.m. and diagrams) represent n = 3 mice.

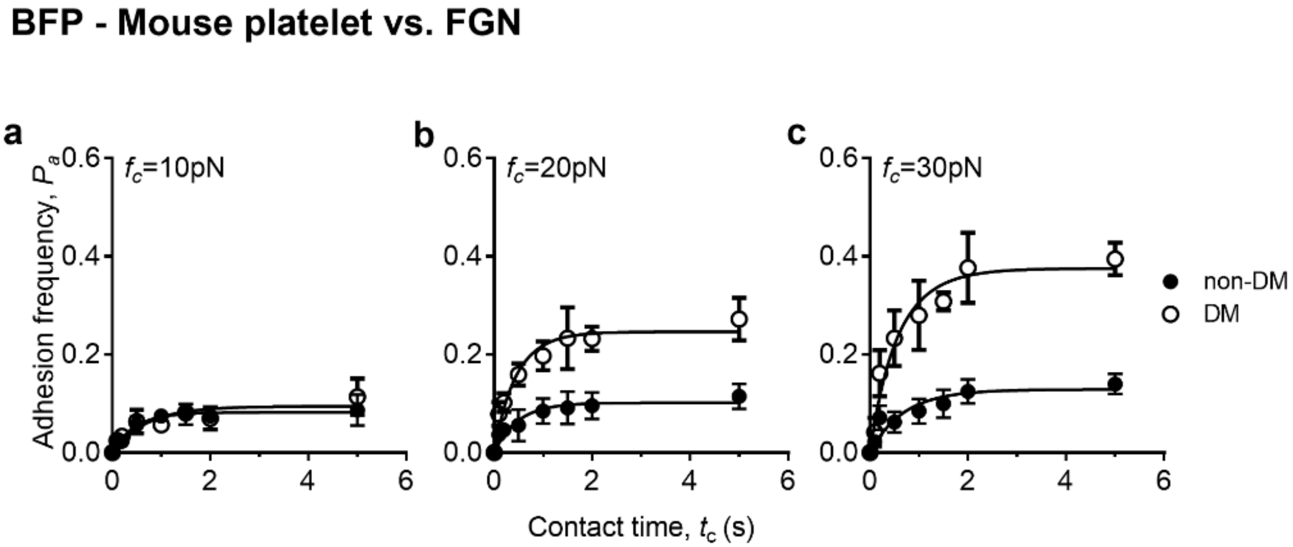

Supplementary Figure 6. 2D kinetics analysis of $\alpha_{IIb}\beta_3$–fibrinogen interaction using BFP [corresponding to Fig. 5a-c]. Non-DM and DM mouse platelet adhesion frequencies (P_a) to FGN coated beads were measured at the indicated BFP contact time (t_c) and three compression forces f_c=10 pN (a), 20 pN (b) and 30 pN (c). P_a vs. t_c plots (points) were fitted with a published equation: $P_a = 1 - \exp\{- m_r m_l A_c k_{on} [1 - \exp(k_{off} t_c)]/k_{off}\}^3$, where m_r and m_l are the respective densities of the receptor and ligand, A_c is the contact area (controlled to be constant), k_{on} and k_{off} are respective 2D on-rate and off-rate. All results represent mean ± s.e.m. of n = 3-4 mice (4 bead-platelet pairs per mouse).

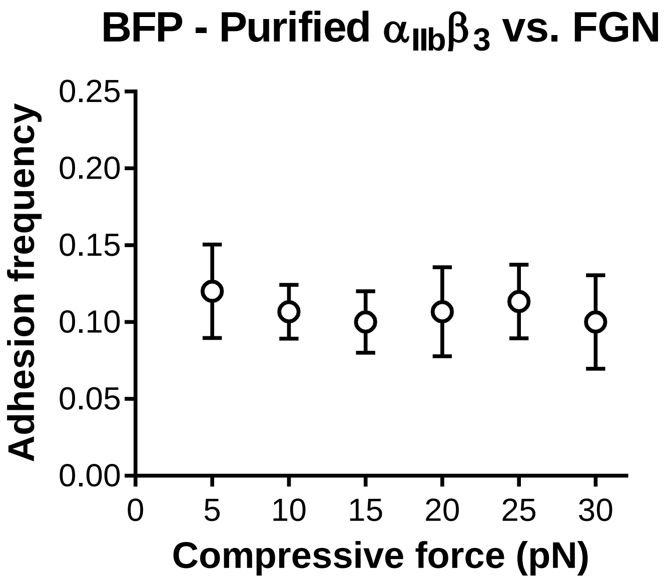

Supplementary Figure 7. Compression forces do not affect purified $\alpha_{IIb}\beta_3$ interaction with fibrinogen [corresponding to Fig. 4e,f]. Purified human $\alpha_{IIb}\beta_3$ coated beads were subjected to 50 BFP cycles against fibrinogen (FGN)-coated beads at the indicated compressive forces. The adhesion frequency was determined. All results represent mean ± s.e.m. of n = 3 independent experiments (4 bead-bead pairs per experiment).

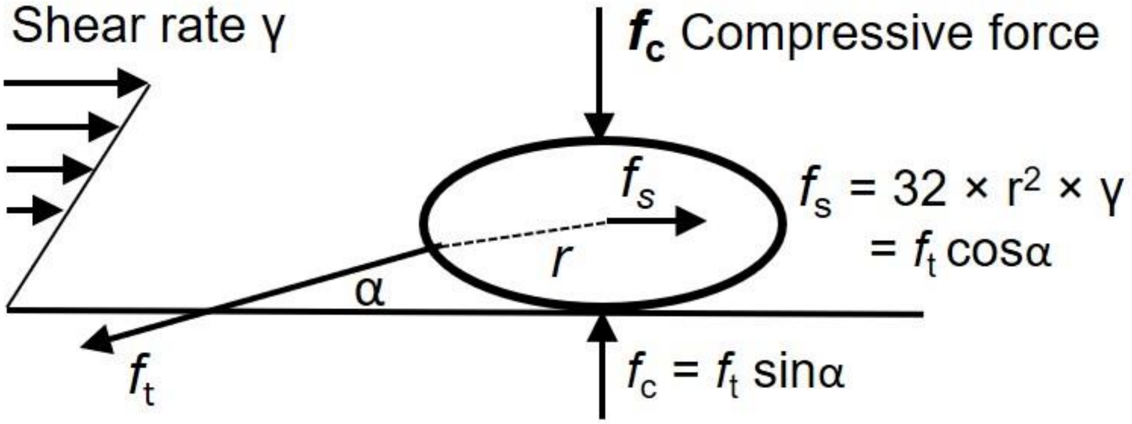

Supplementary Figure 8. Force balance diagram for a platelet tethered to a surface by an adhesive contact at the rear end. The tether force (f_t) is balanced by the hydrodynamic drag force (f_s) and the compressive force (f_c). The hydrodynamic drag force has been estimated as $32 \times r^2 \times \gamma$, where γ is the shear rate of the bulk flow. Considering that the tether force has an angle α, the compressive force and drag force are related to the tether force by $f_c = f_t \sin\alpha$ and $f_s = f_t \cos\alpha$.

SUPPLEMENTARY REFERENCES:

1 Nesbitt, W. S., Tovar-Lopez, F. J., Westein, E., Harper, I. S. & Jackson, S. P. A multimode-TIRFM and microfluidic technique to examine platelet adhesion dynamics. *Methods Mol. Biol.* **1046**, 39-58, doi:10.1007/978-1-62703-538-5_3 (2013).

2 Maxwell, M. J. *et al.* SHIP1 and Lyn Kinase Negatively Regulate Integrin alpha IIb beta 3 signaling in platelets. *J Biol Chem* **279**, 32196-32204, doi:10.1074/jbc.M400746200 (2004).

3 Ju, L., Chen, Y., Rushdi, M. N., Chen, W. & Zhu, C. Two-Dimensional Analysis of Cross-Junctional Molecular Interaction by Force Probes. *Methods Mol Biol* **1584**, 231-258, doi:10.1007/978-1-4939-6881-7_15 (2017).

4 Chen, Y. *et al.* Fluorescence Biomembrane Force Probe: Concurrent Quantitation of Receptor-ligand Kinetics and Binding-induced Intracellular Signaling on a Single Cell. *J Vis Exp*, doi:10.3791/52975 (2015).

5 Nesbitt, W. S., Harper, I. S., Schoenwaelder, S. M., Yuan, Y. & Jackson, S. P. A live cell micro-imaging technique to examine platelet calcium signaling dynamics under blood flow. *Methods Mol. Biol.* **788**, 73-89, doi:10.1007/978-1-61779-307-3_6 (2012).

6 Hughan, S. C. *et al.* Dok-2 adaptor protein regulates the shear-dependent adhesive function of platelet integrin alphaIIbbeta3 in mice. *J Biol Chem* **289**, 5051-5060, doi:10.1074/jbc.M113.520148 (2014).

7 Yago, T. *et al.* Catch bonds govern adhesion through L-selectin at threshold shear. *Journal of Cell Biology* **166**, 913-923, doi:10.1083/jcb.200403144 (2004).

8 Yago, T. *et al.* Platelet glycoprotein Ibα forms catch bonds with human WT vWF but not with type 2B von Willebrand disease vWF. *J Clin Invest*, doi:10.1172/JCI35754 (2008).
