# Supplementary material for: Compression force sensing regulates integrin αIIbβ3 adhesive function on diabetic platelets
Source: Nat Commun. 2018 Mar 14;9:1087. doi: 10.1038/s41467-018-03430-6 (PMC5852038; doi:10.1038/s41467-018-03430-6)
Supplement: Supplementary file 2 — Description of Additional Supplementary Files [file 41467_2018_3430_MOESM2_ESM.pdf]

Description of Additional Supplementary Files

File Name: Supplementary Movie 1
Description: **Diabetes enhances platelet thrombi in a needle *in situ* thrombosis model [corresponding to Fig. 1c,d].** In control (non-DM) and diabetic (DM) mice, thrombus formation was monitored following needle tip insertion (needle *in situ* thrombosis model), as described under 'Methods'. This movie of DIC intravital microscopy demonstrates exaggerated platelet aggregation in DM mice relative to non-DM mice, over a 3-min period post-needle insertion.

File Name: Supplementary Movie 2
Description: **PI3Kβ regulates the enhanced thrombotic response in diabetic mice *in vivo* [corresponding to Fig. 7c-f].** Diabetic (DM) wild-type mice pretreated with DMSO (vehicle), the PI3Kβ inhibitor TGX221 (2.5 mg kg^{-1}) or a combination of aspirin and clopidogrel (Asp/Clop, 200 mg kg^{-1}/3 mg kg^{-1}) and DM $PI3Kb^{-/-}$ mice were subject to the needle *in situ* thrombosis model. Thrombus formation in response to needle insertion was monitored using intravital DIC microscopy for 2 min. This movie demonstrates that the diabetes enhanced thrombotic response was abrogated by PI3Kβ deficiency or inhibition, but not by Asp/Clop treatment.
